# Week-Ahead Prediction of High-Risk Drinking Episodes Among Young Adults Using Wearable Biosignals and Psychological Vulnerabilities: Prospective Observational Machine Learning Study

**DOI:** 10.2196/88223

**Published:** 2026-07-10

**Authors:** Jae Seok Kwak, Hae Kook Lee, Sun-Jin Jo, Jun Hyuk Kwon, Sun Jung Kwon, Yena Kim, Haejung Lee

**Affiliations:** 1 Department of Psychiatry College of Medicine The Catholic University of Korea Seocho-gu, Seoul Republic of Korea; 2 Department of Psychiatry The Catholic University of Korea Uijeongbu St. Mary's Hospital Uijeongbu, Gyeonggi-do Republic of Korea; 3 Department of Addiction Studies Graduate School The Catholic University of Korea Bucheon-si, Gyeonggi-do Republic of Korea; 4 Department of Industrial Engineering Kongju National University Cheonan, Chungcheongnam-do Republic of Korea; 5 Department of Counseling Psychology Korea Baptist Theological University and Seminary Yuseong-gu, Daejeon Republic of Korea; 6 AI Healthcare Division PCN Gangnam-gu, Seoul Republic of Korea

**Keywords:** digital phenotyping, high-risk drinking, machine learning, risk prediction, wearable devices

## Abstract

**Background:**

Although machine learning has increasingly been used to predict mental health symptoms and maladaptive behaviors, real-world prediction of addiction-related risk remains limited. Emotional and temperamental vulnerabilities are established correlates of alcohol-related problems, yet few studies have integrated these factors with wearable-derived biosignals in alcohol-risk prediction models.

**Objective:**

This study evaluated whether machine learning models could predict weekly high-risk drinking episodes among young adults with elevated alcohol-use risk by integrating wearable-derived health data with baseline emotional and personality vulnerability indicators.

**Methods:**

In this prospective observational study, adults in their 20s completed weekly self-report surveys and wore Fitbit devices for 4 weeks. Features from week *t* were used to predict the Alcohol Use Disorders Identification Test-Korean version (AUDIT-K)–based high-risk drinking label at week *t*+1. Positive labels were defined using AUDIT-K high-risk drinking cutoffs, with scores of ≥20 for men and ≥10 for women. Extreme gradient boosting (XGBoost) and random forest models were evaluated across self-report-only, wearable-only, and integrated feature sets using 5-fold participant-level grouped cross-validation.

**Results:**

A total of 206 participants contributed 620 week-level observations, of which 85 (13.7%) were labeled as positive high-risk drinking episodes. In participant-level grouped cross-validation, the integrated random forest model showed the most favorable sensitivity-oriented performance, with a mean accuracy of 0.617 (SD 0.078), recall/sensitivity of 0.653 (SD 0.144), area under the receiver operating characteristic curve (ROC AUC) of 0.681 (SD 0.079), and area under the precision-recall curve (PR AUC) of 0.255 (SD 0.090). The integrated XGBoost model achieved an accuracy of 0.670 (SD 0.089), recall/sensitivity of 0.399 (SD 0.174), ROC AUC of 0.651 (SD 0.089), and PR AUC of 0.228 (SD 0.074). Shapley additive explanations analyses indicated that both baseline vulnerability indicators and wearable-derived weekly summaries contributed to model predictions.

**Conclusions:**

Integrating baseline emotional and personality vulnerability indicators with wearable-derived weekly health signals may provide useful information for week-ahead prediction of high-risk drinking episodes. These findings provide preliminary support for wearable-assisted alcohol-risk stratification, although the modest positive predictive performance indicates that external validation and more proximal within-person measures are needed before real-world early-warning or just-in-time adaptive intervention applications.

## Introduction

### Background

According to the 2021 National Mental Health Survey conducted by the Ministry of Health and Welfare, the 12-month prevalence of alcohol use disorder among Korean adults was 2.6%, yet only 2.6% of those individuals reported receiving any treatment during the past year [1]. In addition, data from the Korea Community Health Survey [2] showed that monthly alcohol consumption increased from 53.7% in 2021 to 58.0% in 2023, a rise of 4.3 percentage points. The prevalence of high-risk drinking also increased from 11.0% in 2021 to 13.2% in 2023. Rising rates of alcohol use and high-risk drinking heighten the likelihood of early alcohol misuse and chronic alcohol dependence, underscoring an urgent need for targeted public-health strategies.

The COVID-19 pandemic further exacerbated alcohol-related problems. According to the 2020 Alcohol Industry Information Survey [3], the incidence of home drinking (72.2%) and drinking alone (74.9%) increased substantially. A twin study of adults also indicated that pandemic-related social restrictions increased stress and anxiety, which in turn led to greater alcohol consumption as a coping mechanism [4]. Collectively, these findings, combined with the limited accessibility of in-person treatment during the pandemic, have intensified the emphasis on digital interventions as an alternative means of addressing alcohol-related problems [5].

Research efforts have increasingly focused on addressing alcohol-related problems by leveraging widely accessible technologies such as smartphones and wearable devices, particularly in response to low treatment use, limited-service accessibility, and poor treatment awareness among individuals with alcohol problems [6-8]. Wearable technologies offer notable advantages by continuously monitoring individuals’ behaviors and physiological states in real time, enabling timely and context-sensitive interventions [9]. Moreover, wearing such devices can enhance users’ sense of empowerment, motivation, and accountability [6], suggesting that wearable-supported approaches may contribute positively to reducing alcohol use and improving outcomes among individuals at risk.

A growing body of research has attempted to predict alcohol-related problems at an early stage. One representative line of work uses smartphone sensors and machine learning to detect or forecast drinking and binge-drinking episodes among young adults. Prior studies have demonstrated the feasibility of classifying and predicting high-risk drinking using passively collected indicators, such as accelerometer data, call logs, and digital device use patterns [10]. More recent work has combined extreme gradient boosting (XGBoost)–based prediction models with artificial intelligence (AI) to anticipate same-day binge drinking 1 to 6 hours in advance, with the aim of enabling just-in-time adaptive interventions (JITAIs) [11]. Early studies further established the technical validity of detecting weekend nighttime drinking or heavy drinking using smartphone sensors with high accuracy [12].

In parallel, digital marker–based screening approaches have emerged, including models that estimate alcohol risk from Instagram images and text using neural network analysis [13]. In the neuroimaging domain, multimodal classifiers integrating structural, task-based, and resting-state connectivity data have demonstrated significant accuracy in distinguishing individuals with alcohol dependence [14]. Similarly, a model combining default mode network functional connectivity, neuropsychological indicators, and impulsivity features using random forest algorithms has been reported [15]. Deep-learning classification using electroencephalography signals has also shown high accuracy even with a limited number of channels and small sample sizes, highlighting the promise of noninvasive physiological markers [16].

Taken together, research across digital sensing, neuroimaging, physiological signals, and behavioral data demonstrates a growing potential for machine learning–based early detection and prediction of alcohol-related behaviors. These approaches collectively aim to balance measurement burden, real-time monitoring capability, and generalizability, thereby advancing the development of lightweight and scalable predictive models for use in real-world settings.

Despite their well-established relevance, emotional problems and personality vulnerabilities have been insufficiently explored in AI and machine learning research on alcohol use. Systematic reviews and empirical studies consistently show that psychological factors such as depression [17,18], anxiety [17,19], and stress [20,21], as well as personality-related vulnerabilities including emotional instability [22], harm avoidance [22-25], and impulsivity [22,26,27], are among the most robust predictors of alcohol dependence and problematic drinking. Exploring whether these emotional and personality vulnerabilities interact with real-world physiological rhythms to predict high-risk drinking at an early stage represents a meaningful and necessary research avenue.

Despite the growing body of international research, machine learning studies that use smartphones or wearable devices to predict alcohol-related problems are virtually absent in South Korea. Although national statistics indicate rising prevalence and increasing rates of hazardous drinking, treatment use, and service accessibility remain critically low. Developing machine learning models capable of detecting early indicators of problematic drinking in individuals’ daily lives, therefore, represents a highly significant public-health priority. Accordingly, this study aims to develop a predictive model for high-risk drinking episodes by passively and unobtrusively collecting real-world biodata through wearable devices and integrating these signals with individuals’ emotional and personality vulnerability profiles. This approach seeks to estimate the onset of high-risk alcohol use in advance, enabling timely and personalized preventive intervention.

### Objectives

This study aims to develop machine learning algorithms capable of predicting the risk of emerging alcohol-related problems among high-risk drinkers using easily accessible tools such as smartphones and wearable devices. To achieve this goal, we collected real-time health data (eg, heart rate, activity, and sleep) via a wearable device and gathered self-reported emotional and personality vulnerability measures through a smartphone interface. We then compared the predictive performance of machine learning models trained on (1) self-report psychological data alone, (2) wearable-derived health data alone, and (3) an integrated feature set combining both.

This approach aligns with prior machine learning research demonstrating that predictive accuracy for mental health outcomes, such as emotional distress, stress responses, and depressive symptoms, improves when physiological sensing data and psychological-behavioral data are jointly modeled [28-30]. Accordingly, this study aimed to develop and evaluate machine learning models for week-ahead prediction of Alcohol Use Disorders Identification Test-Korean version (AUDIT-K)–defined high-risk drinking episodes by comparing self-report-only, wearable-only, and integrated feature sets.

## Methods

### Participants

Participants were recruited through Kiwisurvey, an online panel platform operated by Korea Research International, where an announcement for a mobile-based health behavior monitoring study was posted. Individuals who expressed interest and met the eligibility criteria were screened for inclusion. Eligible participants were Korean adults aged 19-29 years residing in the Seoul or Gyeonggi metropolitan areas, with no visual or hearing impairments. To maintain gender balance, a 1:1 sex allocation strategy was applied during recruitment. Additionally, to ensure sufficient representation of high-risk drinkers, at least 30% of the sample was required to meet the AUDIT-K threshold for hazardous drinking (≥10 for men and ≥6 for women). This recruitment criterion was distinct from the prediction outcome label, which was defined using the AUDIT-K high-risk drinking threshold.

Two offline orientation sessions were held on June 24 and July 1, 2023, and participants were asked to attend one of the sessions. During these sessions, researchers provided detailed instructions regarding study procedures, wearable device setup, and informed consent requirements. A total of 209 individuals provided written informed consent and were enrolled in the study. Of these, 206 participants completed the full 4-week monitoring period and were included in the final analyses. Two participants withdrew consent, and one was excluded due to missing data. Participants received an incentive equivalent to approximately US $100 upon completing the 4-week follow-up period.

### Measures and Procedure

After providing written informed consent, participants received a baseline survey link via their personal smartphones and completed a series of self-report questionnaires. Baseline assessments included sociodemographic characteristics such as gender, age, education level, monthly personal allowance, and residential status. Participants also completed the AUDIT-K at baseline and once weekly for 4 consecutive weeks to assess alcohol-use risk.

The AUDIT-K is a validated screening instrument based on the *International Classification of Diseases, Tenth Revision* diagnostic criteria that assesses alcohol use disorder risk and associated drinking problems. The Korean version consists of 10 items measuring drinking frequency and quantity, symptoms of dependence, and alcohol-related problems [31]. Validation studies have established AUDIT-K cutoff scores of ≥10 for men and ≥6 for women to classify hazardous drinking and ≥20 for men and ≥10 for women to classify high-risk drinking. In this study, the hazardous drinking cutoff was used only for recruitment enrichment, whereas the high-risk drinking cutoff was used to define the binary prediction outcome. The measure demonstrated excellent test-retest reliability in prior validation research (*r*=0.929). In this study, internal consistency at baseline was acceptable (Cronbach α=0.794).

Baseline and week-4 surveys included the Patient Health Questionnaire-9 (PHQ-9), the Generalized Anxiety Disorder scale (GAD-7), and the Perceived Stress Scale-10 (PSS-10). The PHQ-9 demonstrated excellent internal consistency at the time of development (Cronbach α=0.89) [32]. A Korean validation study [33] recommended a cutoff score of 9, reporting strong reliability (Cronbach α=0.95); in this study, internal consistency was acceptable (Cronbach α=0.845). The GAD-7 similarly showed high reliability in its original development (Cronbach α=0.92) [34]. The Korean validated version [35] also demonstrated strong internal consistency (Cronbach α=0.92), and reliability in this sample was high (Cronbach α=0.906). Both the PHQ-9 and GAD-7 assess subjective symptoms experienced over the past 2 weeks using a Likert scale ranging from 0 (“not at all”) to 3 (“nearly every day”). Higher total scores indicate greater severity of depressive or anxiety symptoms, respectively.

We used the Korean validated version of the scale, which measures the degree to which individuals perceive their lives as stressful and comprises 2 factors, negative perception and positive perception, rated on a 5-point Likert scale [36]. Higher total scores indicate greater perceived stress, with possible scores ranging from 0 to 40. Established interpretive ranges categorize scores of 14-16 as mild stress, 17-18 as moderate stress, and ≥19 as severe stress. The original PSS-10 demonstrated strong internal consistency (Cronbach α=0.84-0.86), and reliability in this study was also high (Cronbach α=0.815).

Personality vulnerabilities were assessed using the Personality Vulnerability Scale, a measure developed by the Korean Psychological Association [37] based on transdiagnostic approaches. The scale was designed to identify and screen for dispositional psychological risk factors, including cognitive, emotional, and behavioral processes, that interact with stressful environments to trigger or maintain various mental health problems. The original instrument consists of 58 items rated on a 4-point Likert scale (0=“not at all” to 3=“very much”), organized into 3 overarching domains (emotional and impulse regulation, information processing, and interpersonal/social functioning) and 8 subcomponents.

For the purposes of this study, we extracted 3 subscales known to be closely associated with risky alcohol use: emotional instability, impulsivity, and harm avoidance. Internal consistency at baseline was high for all subscales (Cronbach α=0.891 for emotional instability, 0.808 for impulsivity, and 0.870 for harm avoidance). Reliability in the current dataset also demonstrated excellent internal consistency (Cronbach α=0.870, 0.759, and 0.851, respectively).

All questionnaire data, including baseline and follow-up assessments, were collected via a smartphone-accessible web-based survey platform. Physiological and behavioral data—such as sleep, heart rate, and step count—were passively and continuously recorded in real time using a wearable device (Fitbit Charge 5; Fitbit Inc).

### Statistical Analysis

Supervised classification models were applied to predict a binary outcome variable aggregated at the weekly level. The dependent variable, weekly high-risk drinking episode, was defined using the AUDIT-K high-risk drinking cutoffs, with positive cases coded as ≥20 for men and ≥10 for women. Predictor variables consisted of self-report measures (depression, anxiety, stress, and personality vulnerabilities) and wearable-derived features (summarized metrics of heart rate, activity, and sleep). Missing data were handled according to predefined rules. The full list of features included in the analysis is presented in Table 1.

For psychological predictors in the primary models, only baseline scores of emotional and personality vulnerability indicators were included. These included depression, anxiety, perceived stress, emotional instability, impulsivity, and harm avoidance, and were treated as baseline vulnerability indicators rather than time-varying weekly predictors. Accordingly, these variables were interpreted as between-person vulnerability indicators, whereas week-to-week variation in the prediction task was primarily captured by wearable-derived weekly summaries.

Preliminary exploratory analyses examined whether adding repeated weekly self-report symptom scores improved week-ahead prediction. These weekly self-report scores referred to repeated PHQ-9, GAD-7, and PSS-10 assessments collected during follow-up and paired with the subsequent week’s high-risk drinking label when available. These repeated weekly self-report models were treated as exploratory analyses and were not retained as the final primary modeling approach because they differed from the primary models in feature definition, available feature-label pairs, missing-data structure, and conceptual interpretation. In the primary models, self-report psychological and personality measures were conceptualized as baseline vulnerability indicators rather than rapidly changing weekly predictors. This decision was based on the theoretical nature and assessment time frames of the PHQ-9, GAD-7, PSS-10, and personality vulnerability measures, which are intended to capture symptom severity or relatively stable vulnerability profiles over specified recent periods rather than short-term week-to-week fluctuations. Therefore, the primary modeling framework fixed these self-report vulnerability indicators at baseline and focused on whether week-varying wearable-derived summaries could improve the prediction of the following week’s high-risk drinking label. Because these exploratory models differed from the primary models in feature definition, available feature-label pairs, and missing-data structure, their performance estimates should not be directly compared with the primary model results reported in the Results section. Details are provided in Multimedia Appendix 1.

Wearable-derived features were constructed through a multistep aggregation process. Raw Fitbit-derived heart rate, activity, calorie expenditure, distance, and sleep data were first processed at their available sampling resolution and transformed into minute-level, hour-binned, daily, or sleep episode–level summaries, depending on the type of signal. These intermediate summaries were then aggregated to the weekly level to create the final model features. Thus, variables such as steps_mean and calories_mean represent weekly summaries of minute-level activity or energy-expenditure metrics, whereas variables such as avg_heart_rate_1hour and total_calories represent weekly summaries of hour-binned metrics. Sleep-related variables, such as total_sleep_duration_weekly_avg and sleep_fragmentation_weekly_avg, represent weekly averages of daily or sleep-episode-level sleep summaries**.** The resulting weekly wearable-derived features were paired with baseline psychological variables to predict the high-risk drinking label at week *t*+1 from characteristics observed during week *t*.

**Table 1:** Variables used in machine learning models for predicting weekly high-risk drinking episodes.

Category and variable			Description
**Dependent variable**			
	y	Weekly alcohol episode (1=yes, 0=no; AUDIT-K^a^: male ≥20, female ≥10)	
**Psychometric data**			
	Emotional instability	Total score for emotional instability at baseline	
	Harm avoidance	Total score for harm avoidance at baseline	
	Impulsivity	Total score for impulsivity at baseline	
	PHQ^b^_mean	Mean score for depressive items at baseline	
	PHQ_std	Standard deviation for depressive items at baseline	
	GAD^c^_mean	Mean score for anxiety items at baseline	
	GAD_std	Standard deviation for anxiety items at baseline	
	PSS^d^_mean	Mean score for stress items at baseline	
	PSS_std	Standard deviation for stress items at baseline	
**Physiological data (wearable)**			
	heart_rate_mean	Weekly mean heart rate	
	heart_rate_std	Weekly standard deviation for heart rate	
	heart_rate_max	Weekly maximum heart rate	
	heart_rate_min	Weekly minimum heart rate	
	steps_mean	Weekly mean for minute-level step count	
	steps_std	Weekly standard deviation for minute-level step count	
	steps_max	Weekly maximum for minute-level step count	
	steps_min	Weekly minimum for minute-level step count	
	distance_mean	Weekly mean for minute-level distance (m)	
	distance_std	Weekly standard deviation for minute-level distance	
	distance_max	Weekly maximum for minute-level distance	
	distance_min	Weekly minimum for minute-level distance	
	calories_mean	Weekly mean for minute-level calories burned	
	calories_std	Weekly standard deviation for minute-level calories burned	
	calories_max	Weekly maximum for minute-level calories burned	
	calories_min	Weekly minimum for minute-level calories burned	
	avg_heart_rate_1hour	Weekly mean for hour-binned average heart rate	
	std_heart_rate_1hour	Weekly mean for hour-binned heart rate standard deviation	
	total_steps	Weekly mean for hour-binned total steps	
	total_distance	Weekly mean for hour-binned total distance	
	total_calories	Weekly mean for hour-binned total calories burned	
	active_minutes	Weekly mean for hour-binned minutes with active_rate ≥2	
	ratio_light_sleep	Weekly mean for hour-binned light-sleep ratio	
	ratio_sleep	Weekly mean for hour-binned total-sleep ratio	
	ratio_deep_sleep	Weekly mean for hour-binned deep-sleep ratio	
	movement_burst_count	Weekly count for ≥3-minute activity bursts within 10-minute windows	
	resting_duration	Weekly summary of the longest continuous resting duration with active_rate=1 (min)	
	step_zero_streak	Weekly summary for continuous inactivity duration with steps=0	
	total_sleep_duration_weekly_avg	Weekly average for daily total sleep duration	
	sleep_fragmentation_weekly_avg	Weekly average number of daily sleep-stage transitions	
	max_hr_spike_weekly_avg	Weekly average number of ≥10 bpm heart-rate spikes per minute	
	hr_variability_window_weekly_avg	Weekly average for window-based heart rate variability (SD)	
	nighttime_hr_avg_weekly_avg	Weekly average for mean heart rate between 00:00 and 06:00	
	unexpected_activity_at_night_occurred	Weekly indicator of whether active_rate ≥3 occurred between 00:00 and 06:00	
	nocturnal_walking_occurred	Weekly indicator of whether steps or distance >0 occurred during sleep	
	resting_high_calorie_occurred	Weekly indicator of whether high calorie expenditure occurred during inactivity (steps=0 and active_rate=1)	

^a^AUDIT-K: Alcohol Use Disorders Identification Test-Korean version.

^b^PHQ: Patient Health Questionnaire-9.

^c^GAD: Generalized Anxiety Disorder-7.

^d^PSS: Perceived Stress Scale-10.

**Figure 1:**
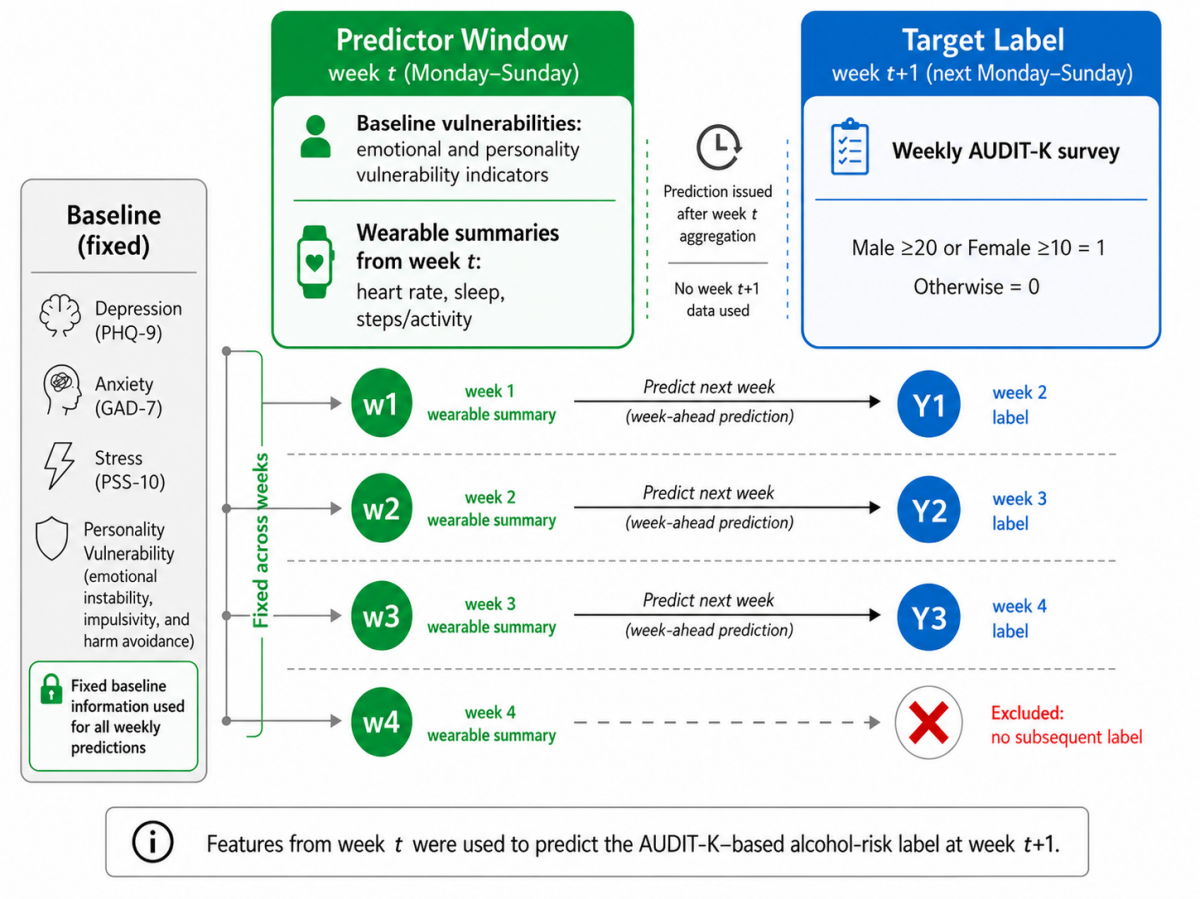
Week-ahead prediction framework and feature-label alignment. AUDIT-K: Alcohol Use Disorders Identification Test-Korean version; GAD-7: Generalized Anxiety Disorder-7; PHQ-9: Patient Health Questionnaire-9; PSS-10: Perceived Stress Scale-10.

To clarify temporal alignment, each participant’s data were organized into Monday to Sunday calendar weeks. Wearable-derived signals collected during week *t* were aggregated only after the week *t* observation window was complete. Weekly survey responses were collected following each calendar week and were used to define the alcohol-risk label for that corresponding week. Prediction was conceptualized as occurring after week *t* feature aggregation but before week *t*+1 outcome information was available; therefore, no information from the target week was used to construct predictors for that target label. Figure 1 illustrates the temporal alignment used to construct week-ahead feature-label pairs. Model evaluation was conducted separately using participant-level grouped cross-validation. The analytical task was defined as a week-ahead binary classification problem in which features aggregated at week *t* were used to predict the positive label at week *t*+1. For each participant, week indices were chronologically ordered, and (t, t+1) feature-label pairs were generated only when the label for week *t*+1 was observed. Observations from the final week, for which no subsequent label existed, were excluded from model training and evaluation (Figure 1). Wearable signals and self-report indicators were summarized from daily raw data into weekly statistics, including means, SDs, quantiles, and proportional measures (eg, proportion of time spent in specific sleep stages). Temporal context variables, such as weekday/weekend indicators and the number of valid observation days per week, were also included in the feature set. Missing data were handled according to the nature and distribution of each variable. Weeks with insufficient within-week observations were excluded entirely, whereas simple imputation or model-tolerant missingness strategies were applied when appropriate. All scaling and imputation procedures were derived exclusively from statistics calculated within each training fold (eg, fold-specific mean and SD) to strictly prevent information leakage into validation folds.

Model comparisons were conducted across three feature configurations: (1) self-report features only, (2) wearable-derived health features only, and (3) an integrated feature set combining self-report and wearable-derived health data. Two supervised classifiers, XGBoost and random forest, were implemented. The primary model evaluation framework was a 5-fold participant-level stratified grouped cross-validation. Participant ID was used as the grouping variable, ensuring that all week-level observations from the same participant were assigned to the same fold and did not appear simultaneously in both the training and held-out folds. Label stratification was applied as far as possible to maintain a comparable distribution of positive high-risk drinking labels across folds. The cross-validation split was generated using a fixed random seed of 42, and fixed random seeds were also applied to stochastic model-training procedures to support reproducibility. This procedure was used to prevent subject-level information leakage arising from repeated weekly observations within individuals.

Within each cross-validation iteration, the held-out fold was used only for final performance estimation. All preprocessing steps, including missing-data handling, scaling where applicable, and any feature transformation, were fitted exclusively on the training portion of that fold and then applied to the held-out fold. Class imbalance was addressed within the training portion of each fold using algorithm-specific class weighting rather than oversampling or undersampling. For XGBoost, the positive-class weight was adjusted based on the ratio of negative to positive labels within the training portion of each fold. For random forest, balanced class weights were applied. No oversampling, undersampling, synthetic minority oversampling technique, or synthetic data generation was applied to the held-out fold. Hyperparameter tuning was conducted within the training portion of each fold using internal validation, and the held-out fold was not used for model selection or threshold determination. Details of the Optuna hyperparameter search spaces, number of trials, and key hyperparameter ranges are provided in Multimedia Appendix 2.

Because the intended use of the model was early detection of future high-risk drinking episodes, recall/sensitivity was prespecified as the primary performance metric. Threshold-dependent metrics were calculated using a sensitivity-oriented decision threshold of 0.20 to prioritize early detection while monitoring precision/positive predictive value (PPV). Precision/PPV, recall/sensitivity, negative predictive value (NPV), and *F*_1_-score were calculated at this operating point. Model discrimination was evaluated using the area under the receiver operating characteristic curve (ROC AUC) and the area under the precision-recall curve (PR AUC), which are threshold-independent metrics. Final performance estimates were reported as the mean and SD across the 5 participant-level grouped cross-validation folds. All analyses were conducted in Python using *scikit-learn*, *xgboost*, and *Optuna*.

To improve transparency and completeness of reporting, the Methods and Results sections were reviewed and revised with reference to the TRIPOD+AI (Transparent Reporting of a Multivariable Prediction Model for Individual Prognosis or Diagnosis Plus Artificial Intelligence) reporting guidance for prediction model studies using machine learning methods.

### Ethical Considerations

All study procedures were conducted in accordance with the Declaration of Helsinki. The institutional review board of the College of Medicine, The Catholic University of Korea, approved this study (MC22OIDI0117). All participants attended an in-person orientation session, during which the study objectives and data collection procedures were fully explained, and written informed consent was obtained from all participants prior to enrollment.

To protect participant privacy and confidentiality, identifying information was stored separately from the research data. Online self-report data and wearable-derived biometric data were managed using study registration numbers after direct identifiers were removed. The linkage file connecting personal identifiers with study registration numbers was stored in encrypted form, and access was restricted to designated data-management personnel. Electronic files and computers were password-protected, and physical documents, including consent forms, were stored in double-locked storage at Korea Research International. Unauthorized access, viewing, or modification of the study data was not permitted under the study data management procedures.

Participants received a coffee voucher worth KRW 5000 (approximately US $3.24) after completing the orientation, consent forms, and baseline questionnaires. Participants who completed the study received KRW 100,000 (approximately US $64.71) as a single bank transfer. Consent for collecting bank account information for compensation payment was included in the informed consent form.

## Results

### Participant Characteristics

Table 2 summarizes the general characteristics of the 206 participants included in the analysis. The sample showed a balanced gender distribution, with 102 (49.5%) men and 104 (50.5%) women. Participants were primarily in their mid to late 20s: 129 (62.6%) individuals were aged 25-29 years, and 77 (37.4%) individuals were aged 19-24 years, with a mean age of 25.21 (SD 2.54) years. Regarding educational attainment, 118 (57.3%) participants had completed a university degree, 60 (29.1%) were currently enrolled as undergraduates, 7 (3.4%) were on a leave of absence, 12 (5.8%) held a graduate-level degree, and 9 (4.4%) had a high school education or below. Monthly personal allowance was ≤US $500 for 95 (46.1%) participants and US $501-US $1000 for 74 (35.9%) participants, with an average of US $783 (SD 558), assuming an approximate conversion rate of US $1=KRW 1000. In terms of living arrangements, 115 (55.8%) participants lived with their parents, 82 (39.8%) lived independently, 6 (2.9%) resided in dormitories, and 3 (1.5%) lived with relatives.

**Table 2:** General characteristics of participants (N=206).

Variable and category			Value
**Gender, n (%)**			
	Male	102 (49.5)	
	Female	104 (50.5)	
**Age (years), n (%)**			
	19-24	77 (37.4)	
	25-29	129 (62.6)	
Age (years), mean (SD)			25.21 (2.54)
**Education level, n (%)**			
	High school or below	9 (4.4)	
	Undergraduate (enrolled)	60 (29.1)	
	Undergraduate (on leave)	7 (3.4)	
	Bachelor’s degree	118 (57.3)	
	Graduate school or higher	12 (5.8)	
**Monthly personal allowance (US $)^a^, n (%)**			
	≤500	95 (46.1)	
	501-1000	74 (35.9)	
Monthly personal allowance (US $), mean (SD)			783 (558)
**Housing type, n (%)**			
	Living independently	82 (39.8)	
	Parents’ home	115 (55.8)	
	Dormitory	6 (2.9)	
	Relative’s home	3 (1.5)	

^a^Refers to the monthly allowance an individual uses for personal expenses (eg, transportation, eating out, and cultural activities). For convenience, US $1=KRW 1000 was used for conversion.

**Figure 2:**
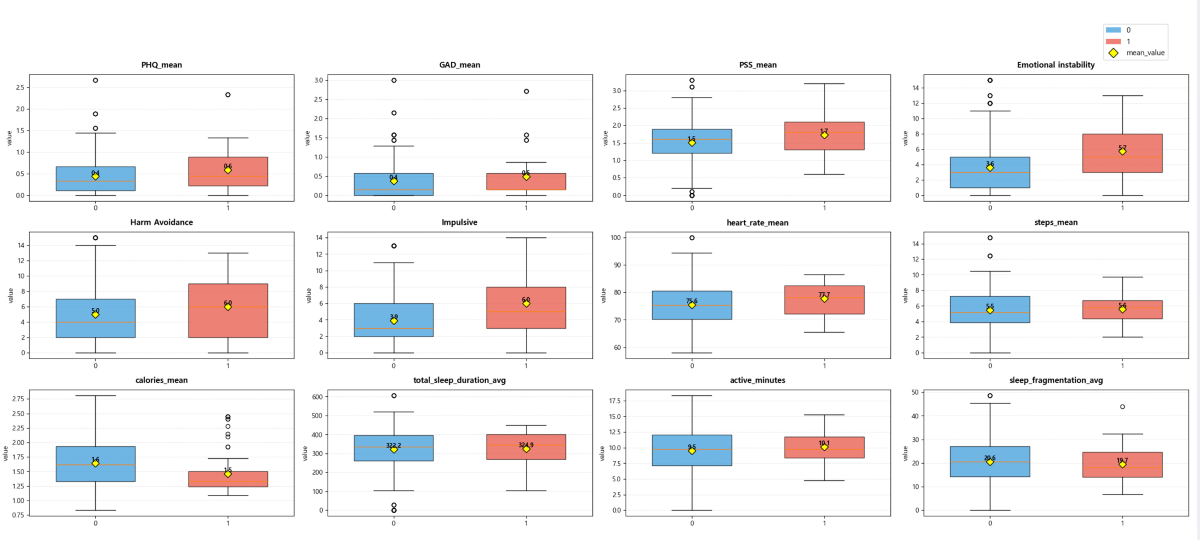
Comparison of psychological, personality, and wearable-derived behavioral indicators between groups. GAD: Generalized Anxiety Disorder-7; PHQ: Patient Health Questionnaire-9; PSS: Perceived Stress Scale-10.

Figure 2 presents group comparisons between the nonepisode group (0) and the episode group (1) across psychological, personality, and wearable-derived physiological and behavioral indicators. The episode group showed higher levels of depressive symptoms (PHQ_mean), anxiety (GAD_mean), and perceived stress (PSS_mean), as well as higher scores on personality vulnerability traits, including emotional instability, harm avoidance, and impulsivity. These patterns suggest that individuals in the episode group were more emotionally vulnerable and exposed to greater negative affective states.

Clear differences were also observed in wearable-derived health metrics. The episode group exhibited a higher average heart rate (heart_rate_mean) but lower average daily steps (steps_mean) and calorie expenditure (calories_mean). Additionally, they demonstrated shorter total sleep duration (total_sleep_duration_avg) and greater sleep fragmentation (sleep_fragmentation_avg). Collectively, these findings indicate that the episode group experienced reduced behavioral stability, heightened physiological arousal, and disruptions in sleep-activity rhythms compared to the nonepisode group.

### Data Overview

A total of 620 week-level observations were obtained from 206 participants across the 4-week monitoring period. Based on the AUDIT-K high-risk drinking thresholds (≥20 for men and ≥10 for women), 85 (13.7%) observations were labeled as positive high-risk drinking episodes, and 535 (86.3%) observations were labeled as negative episodes.

**Figure 3:**
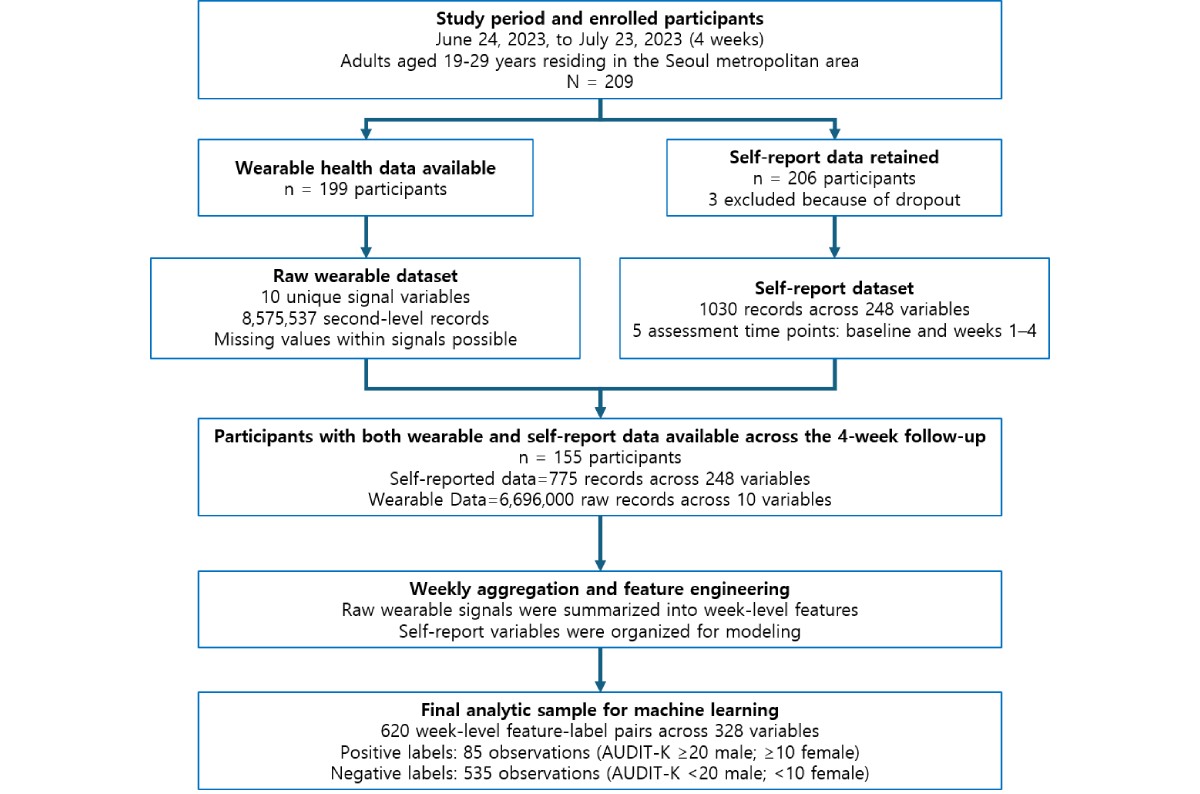
Study sample and derivation of the analytic dataset. AUDIT-K: Alcohol Use Disorders Identification Test-Korean version.

Model development and evaluation were conducted using 5-fold participant-level grouped cross-validation. All week-level observations from the same participant were kept within the same fold to avoid subject-level leakage. Therefore, model performance was estimated from held-out participants rather than from randomly split week-level observations. Within each fold, preprocessing, class-imbalance handling through algorithm-specific class weighting, hyperparameter tuning, and model fitting were performed using only the training portion, and the held-out fold was used exclusively for performance estimation. The overall modeling workflow is summarized in Figure 3. Accordingly, all primary performance estimates were derived from participant-level grouped cross-validation rather than from randomly split week-level observations.

### Performance of High-Risk Drinking Episode Prediction Models

Table 3 summarizes model performance estimated using 5-fold participant-level grouped cross-validation. To prevent information leakage, all preprocessing procedures, class-imbalance correction, hyperparameter tuning, and model fitting were performed exclusively within the training portion of each fold; the held-out fold was used only for final performance estimation. Because the intended application was early detection of future high-risk drinking episodes, threshold-dependent metrics were calculated using a sensitivity-oriented decision threshold of 0.20, while ROC AUC and PR AUC were evaluated as threshold-independent discrimination metrics.

Across the evaluated models, the integrated random forest model showed the most favorable early-detection profile. This model achieved a mean accuracy of 0.617 (SD 0.078), precision/PPV of 0.209 (SD 0.069), recall/sensitivity of 0.653 (SD 0.144), NPV of 0.919 (SD 0.036), *F*_1_-score of 0.315 (SD 0.097), ROC AUC of 0.681 (SD 0.079), and PR AUC of 0.255 (SD 0.090). The integrated XGBoost model showed higher accuracy but lower sensitivity, with a mean accuracy of 0.670 (SD 0.089), precision/PPV of 0.172 (SD 0.039), recall/sensitivity of 0.399 (SD 0.174), NPV of 0.880 (SD 0.059), *F*_1_-score of 0.236 (SD 0.066), ROC AUC of 0.651 (SD 0.089), and PR AUC of 0.228 (SD 0.074).

For models trained on self-report features only, the XGBoost classifier achieved a mean accuracy of 0.396 (SD 0.225), precision/PPV of 0.128 (SD 0.044), recall/sensitivity of 0.630 (SD 0.356), NPV of 0.884 (SD 0.085), *F*_1_-score of 0.203 (SD 0.076), ROC AUC of 0.603 (0.084), and PR AUC of 0.213 (0.023). The random forest classifier showed a mean accuracy of 0.491 (SD 0.057), precision/PPV of 0.150 (SD 0.044), recall/sensitivity of 0.607 (SD 0.221), NPV of 0.882 (SD 0.086), *F*_1_-score of 0.234 (SD 0.061), ROC AUC of 0.569 (SD 0.137), and PR AUC of 0.226 (SD 0.080).

For wearable-derived health features only, the XGBoost model yielded a mean accuracy of 0.727 (SD 0.086), precision/PPV of 0.208 (SD 0.095), recall/sensitivity of 0.393 (SD 0.246), NPV of 0.888 (SD 0.069), *F*_1_-score of 0.260 (SD 0.133), ROC AUC of 0.649 (SD 0.087), and PR AUC of 0.248 (SD 0.070). The random forest model achieved a mean accuracy of 0.601 (SD 0.056), precision/PPV of 0.185 (SD 0.061), recall/sensitivity of 0.581 (SD 0.147), NPV of 0.902 (SD 0.051), *F*_1_-score of 0.278 (SD 0.086), ROC AUC of 0.610 (SD 0.076), and PR AUC of 0.264 (SD 0.107).

Overall, the integrated random forest model showed the highest recall/sensitivity and ROC AUC among the evaluated models, suggesting that combining baseline psychological vulnerability indicators with wearable-derived weekly summaries may improve sensitivity for detecting future high-risk drinking episodes. However, precision/PPV remained modest across models, indicating that the models should be interpreted as preliminary risk-stratification tools rather than stand-alone decision systems.

**Figure 4:**
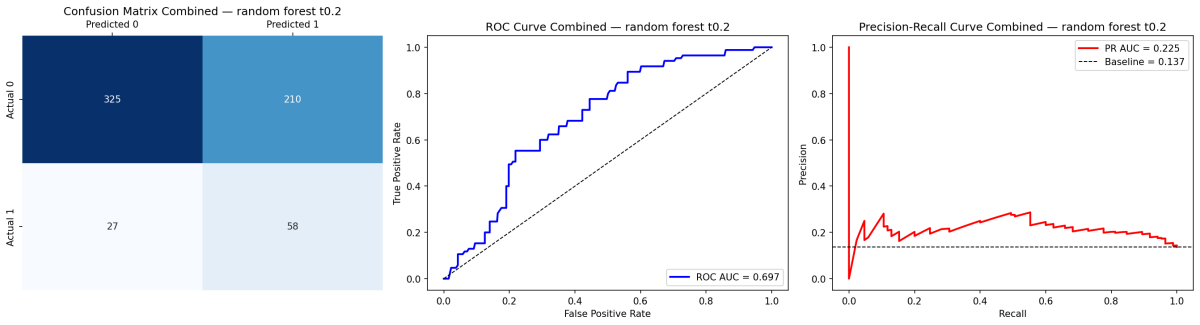
Prediction performance of the integrated extreme gradient boosting model. PR AUC: area under the precision-recall curve; ROC: receiver operating characteristic curve.

**Figure 5:**
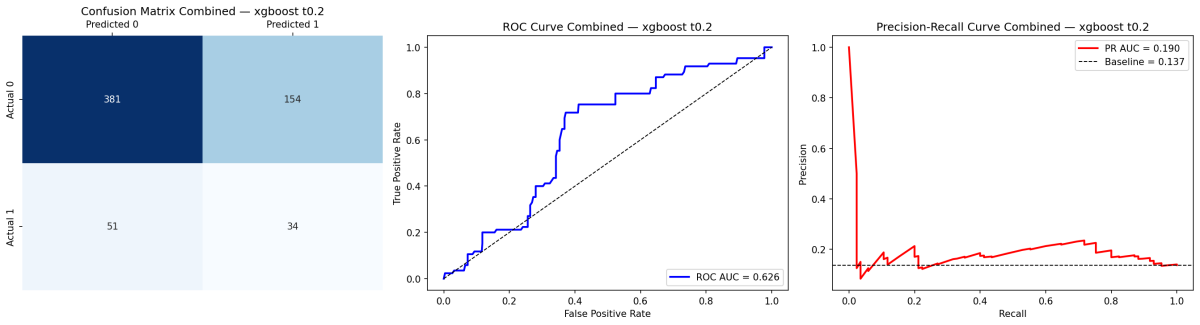
Prediction performance of the integrated random forest model. PR AUC: area under the precision-recall curve; ROC: receiver operating characteristic curve.

The confusion matrices and ROC curves for the integrated XGBoost and random forest models were generated from pooled out-of-fold predictions across participant-level grouped cross-validation folds and are presented in Figures 4 and 5, respectively.

**Table 3:** Machine learning model performance for week-ahead prediction of high-risk drinking episodes^a^.

Feature set	Model	Accuracy, mean (SD)	Precision/PPV^b^, mean (SD)	Recall/sensitivity, mean (SD)	NPV^c^, mean (SD)	*F*_1_-score, mean (SD)	ROC AUC^d^, mean (SD)	PR AUC^e^, mean (SD)
Self-report only	XGBoost^f^	0.396 (0.225)	0.128 (0.044)	0.630 (0.356)	0.884 (0.085)	0.203 (0.076)	0.603 (0.084)	0.213 (0.023)
Self-report only	Random forest	0.491 (0.057)	0.150 (0.044)	0.607 (0.221)	0.882 (0.086)	0.234 (0.061)	0.569 (0.137)	0.226 (0.080)
Health data only	XGBoost	0.727 (0.086)	0.208 (0.095)	0.393 (0.246)	0.888 (0.069)	0.260 (0.133)	0.649 (0.087)	0.248 (0.070)
Health data only	Random forest	0.601 (0.056)	0.185 (0.061)	0.581 (0.147)	0.902 (0.051)	0.278 (0.086)	0.610 (0.076)	0.264 (0.107)
Integrated	XGBoost	0.670 (0.089)	0.172 (0.039)	0.399 (0.174)	0.880 (0.059)	0.236 (0.066)	0.651 (0.089)	0.228 (0.074)
Integrated	Random forest	0.617 (0.078)	0.209 (0.069)	0.653 (0.144)	0.919 (0.036)	0.315 (0.097)	0.681 (0.079)	0.255 (0.090)

^a^Values are presented as mean (SD) across 5 participant-level grouped cross-validation folds. Threshold-dependent metrics were calculated using a decision threshold of 0.20. Class imbalance was addressed using algorithm-specific class weighting within the training portion of each fold. Area under the receiver operating characteristic curve and area under the precision-recall curve are threshold-independent discrimination metrics. Precision is equivalent to positive predictive value, and recall is equivalent to sensitivity.

^b^PPV: positive predictive value.

^c^NPV: negative predictive value.

^d^ROC AUC: area under the receiver operating characteristic curve.

^e^PR AUC: area under the precision-recall curve.

^f^XGBoost: extreme gradient boosting.

### Model Explainability Using Shapley Additive Explanations

**Figure 6:**
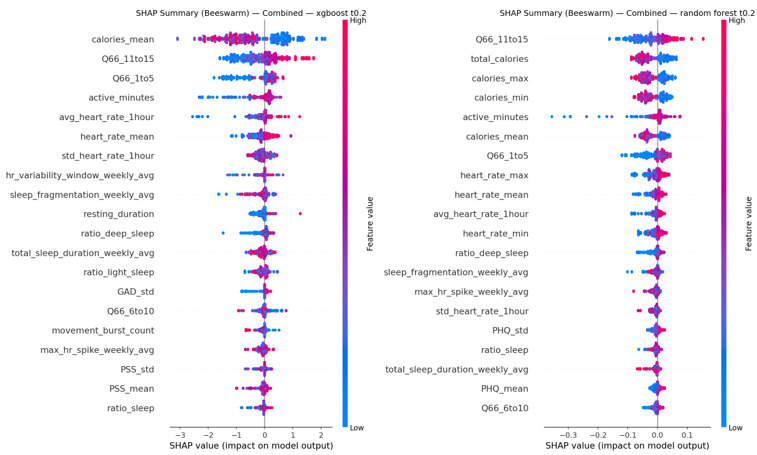
Shapley additive explanations (SHAP) summary plots for the integrated models: extreme gradient boosting (XGBoost; left) and random forest (right).

**Figure 7:**
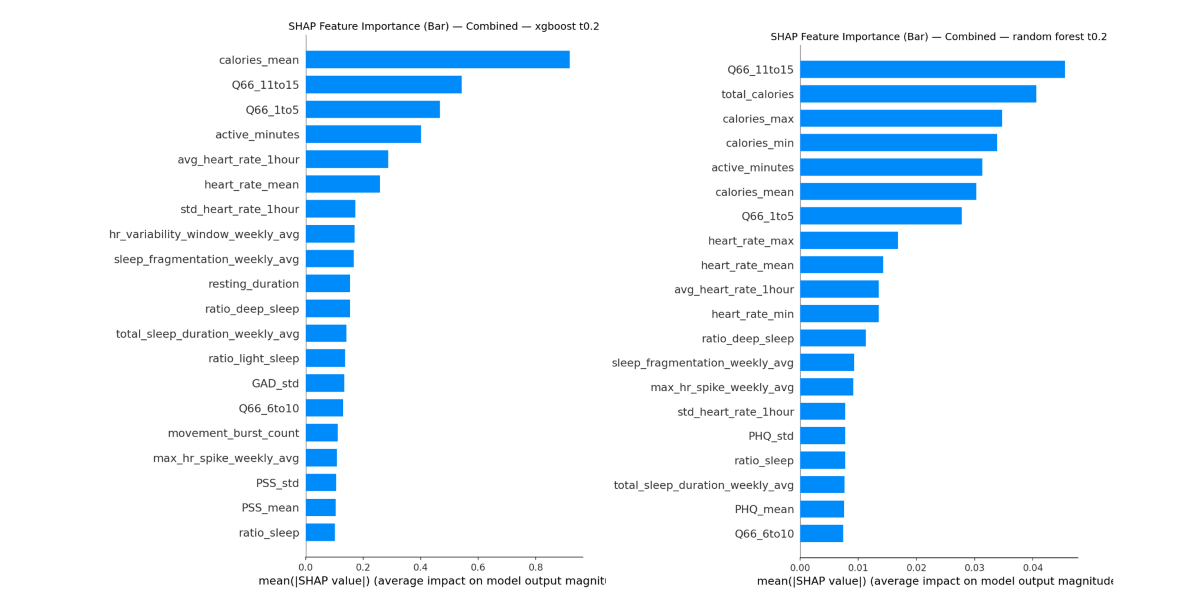
Top 20 features ranked by mean absolute Shapley additive explanations (SHAP) values: integrated extreme gradient boosting (XGBoost; left) and integrated random forest (right).

We examined which features contributed most strongly to the performance of each machine learning model. Shapley additive explanations (SHAP) summary plots and bar plots visually illustrate the variables that contributed most to model predictions (Figures 6 and 7 and Table 4). Because SHAP values quantify feature contributions to model output rather than causal effects, the identified variables were interpreted as predictive correlates rather than physiological precursors or causal mechanisms.

**Table 4:** Top-ranked Shapley additive explanations (SHAP) feature importance scores for the integrated extreme gradient boosting (XGBoost) and random forest models^a^.

Rank and feature			SHAP feature importance score
**XGBoost**			
	1. Mean calorie expenditure (calories_mean)	0.918	
	2. Impulsivity (Q66_11to15)	0.543	
	3. Emotional instability (Q66_1to5)	0.467	
	4. Active minutes (active_minutes)	0.401	
	5. Average hourly heart rate (avg_heart_rate_1hour)	0.287	
	6. Mean heart rate (heart_rate_mean)	0.259	
	7. Hourly heart rate variability (std_heart_rate_1hour)	0.174	
	8. Weekly heart rate variability (hr_variability_window_weekly_avg)	0.170	
	9. Sleep fragmentation (sleep_fragmentation_weekly_avg)	0.168	
	10. Resting duration (resting_duration)	0.155	
	11. Ratio of deep sleep (ratio_deep_sleep)	0.155	
	12. Total sleep duration (total_sleep_duration_weekly_avg)	0.142	
	13. Ratio of light sleep (ratio_light_sleep)	0.138	
	14. Anxiety variability (GAD_std)	0.135	
	15. Harm avoidance (Q66_6to10)	0.131	
**Random forest feature**			
	1. Impulsivity (Q66_11to15)	0.045	
	2. Total calories (total_calories)	0.041	
	3. Maximum calories (calories_max)	0.035	
	4. Minimum calories (calories_min)	0.034	
	5. Active minutes (active_minutes)	0.031	
	6. Mean calorie expenditure (calories_mean)	0.030	
	7. Emotional instability (Q66_1to5)	0.028	
	8. Maximum heart rate (heart_rate_max)	0.017	
	9. Mean heart rate (heart_rate_mean)	0.014	
	10. Average hourly heart rate (avg_heart_rate_1hour)	0.014	
	11. Minimum heart rate (heart_rate_min)	0.014	
	12. Ratio of deep sleep (ratio_deep_sleep)	0.011	
	13. Sleep fragmentation (sleep_fragmentation_weekly_avg)	0.009	
	14. Maximum heart rate spikes (max_hr_spike_weekly_avg)	0.009	
	15. Hourly heart rate variability (std_heart_rate_1hour)	0.008	

^a^The table presents the top 15 features ranked by SHAP feature importance scores in the integrated models combining self-report and wearable-derived health features. SHAP feature importance scores were calculated as the average absolute SHAP values across observations for each feature and were used to rank the relative contribution of predictors within each model. SHAP values indicate relative feature contributions to model output and should be interpreted as predictive correlates rather than causal effects.

To interpret the integrated XGBoost model, we conducted SHAP analyses to assess feature importance and contribution patterns. Based on the SHAP summary plot and mean absolute SHAP values, the most influential features included mean calorie expenditure (calories_mean), impulsivity (Q66_11to15), emotional instability (Q66_1to5), active minutes, average heart rate per hour (avg_heart_rate_1hour), and mean heart rate (heart_rate_mean). These findings indicate that the integrated model relied on both baseline vulnerability indicators and wearable-derived weekly summaries when classifying next-week high-risk drinking labels.

Among baseline psychological indicators, impulsivity and emotional instability demonstrated prominent contributions to model predictions. Among wearable-derived features, calorie expenditure, active minutes, and heart-rate summaries contributed prominently to the model output. This pattern suggests that baseline emotional and personality vulnerability indicators provided between-person risk information, whereas wearable-derived summaries contributed week-varying behavioral and physiological information. In the integrated XGBoost model, lower values of calorie expenditure tended to contribute positively to model output for high-risk classification, whereas higher values of impulsivity and emotional instability generally contributed toward higher predicted risk. These patterns should be interpreted as model-identified predictive correlates associated with the classification of subsequent high-risk drinking labels.

To interpret the integrated random forest model, we conducted SHAP analyses to identify the features that contributed most strongly to next-week predictions of high-risk drinking episodes. Based on the SHAP summary plot and rankings of mean absolute SHAP values, the most influential variables included impulsivity (Q66_11to15), total calories, maximum calories, minimum calories, active minutes, mean calories, emotional instability (Q66_1to5), maximum heart rate, mean heart rate, and average heart rate per hour.

The random forest model similarly showed that both baseline vulnerability indicators and wearable-derived activity, heart rate, sleep, or calorie-related summaries contributed to prediction outcomes. Among baseline psychological variables, impulsivity and emotional instability demonstrated consistent contributions. Among wearable-derived variables, calorie-related indices, active minutes, and heart rate indicators were prominent contributors. The integrated random forest model showed a particularly strong contribution of impulsivity, followed by calorie-related wearable features. These findings indicate that SHAP-identified features helped explain model classification patterns, but they do not establish causal pathways or temporally specific physiological mechanisms.

Because this was an observational sensing study, wearable-derived predictors may also reflect unmeasured contextual or behavioral factors, including physical activity routines, sleep debt, acute illness, academic or work schedules, weekend social activity, shift work, device wear time, and consumer-device estimation error. Therefore, SHAP-identified features should be interpreted as predictive correlates that require validation in future studies using more granular contextual, behavioral, and physiological data.

## Discussion

### Principal Findings

This study developed machine learning models that integrate psychological and personality vulnerability factors with wearable-derived physiological and behavioral signals to predict weekly high-risk drinking episodes among young adults. Several key findings emerged.

First, the integrated models combining biosignals and psychological-personality vulnerabilities showed a modest but meaningful early-detection profile under 5-fold participant-level grouped cross-validation compared with models using either feature set alone. The integrated XGBoost model achieved a mean accuracy of 0.670 (SD 0.089) and a mean ROC AUC of 0.651 (SD 0.089), while the integrated random forest model achieved a mean accuracy of 0.617 (SD 0.078) and a ROC AUC of 0.681 (SD 0.079). The corresponding PR AUC values were mean 0.228 (SD 0.074) for XGBoost and mean 0.255 (SD 0.090) for random forest. These cross-validated estimates suggest that the integrated models provided modest but useful week-ahead predictive information for high-risk drinking episodes under participant-level grouped validation. In particular, the integrated random forest model showed the most favorable sensitivity-oriented performance, with a mean recall/sensitivity of 0.653 (SD 0.144) and a mean NPV of 0.919 (SD 0.036). However, precision/PPV remained modest at a mean of 0.209 (SD 0.069), indicating that the model should be interpreted as a preliminary risk-stratification tool rather than a stand-alone decision system.

These findings are broadly consistent with previous machine learning and AI-based studies addressing alcohol dependence and substance-use risk. For example, a study detecting substance-use risk from Instagram images reported an ROC AUC of 0.65 [13], and a multimodal neuroimaging classifier for chronic alcohol dependence achieved an accuracy of 79.3% [14]. Research predicting high-risk drinking patterns among medical students reported an area under the curve of 0.738 [38]. Smartphone sensor–based studies forecasting heavy drinking showed a wider performance range, with accuracies from 94.3% to 96.6% [11,12]. However, direct comparison across studies should be made cautiously because prediction horizons, outcome definitions, data modalities, validation schemes, operating thresholds, and participant-level leakage controls differ substantially across studies.

In comparison, a distinctive strength of this study lies in its integration of behavioral biosignals with psychological and personality vulnerabilities that are theoretically and empirically central to alcohol-use problems. Rather than relying on a single modality or purely behavioral data, the present models incorporated emotional vulnerability, including depression, anxiety, and perceived stress, and personality traits such as emotional instability and impulsivity. This multimodal approach may improve week-ahead risk estimation by combining stable vulnerability profiles with week-varying physiological and behavioral information. The integrated model should therefore be interpreted as combining 2 distinct sources of predictive information: stable between-person vulnerability captured by baseline psychological and personality indicators, and week-varying behavioral and physiological information captured by wearable-derived summaries. Accordingly, the model’s week-ahead performance does not necessarily imply that baseline psychological variables captured short-term within-person psychological change. Rather, these variables likely contributed to identifying individuals with higher underlying vulnerability, while wearable-derived weekly features provided the time-varying component of the prediction.

Examining the most influential predictors showed that both baseline vulnerability indicators and wearable-derived weekly summaries contributed to model prediction. In the integrated XGBoost model, the features with the highest mean absolute SHAP values included mean calorie expenditure, impulsivity, emotional instability, active minutes, average heart rate per hour, and mean heart rate. In the integrated random forest model, the highest-ranking SHAP features included impulsivity, total calories, maximum calories, minimum calories, active minutes, mean calories, emotional instability, maximum heart rate, mean heart rate, and average heart rate per hour.

Across both integrated models, impulsivity and emotional instability emerged as prominent baseline vulnerability indicators, while calorie-related metrics, active minutes, and heart-rate summaries were consistently identified among the most influential wearable-derived features. This pattern suggests that the models combined relatively stable between-person vulnerability information with week-level behavioral and physiological summaries when estimating the likelihood of a subsequent high-risk drinking label. The convergence of feature rankings across the 2 algorithms also supports the relevance of integrating psychological vulnerability profiles with passive wearable signals for week-ahead alcohol-risk prediction. Although these SHAP-identified features helped explain the models’ classification patterns, they should be interpreted as predictive correlates rather than causal mechanisms. Because this was an observational sensing study, activity-, calorie-, and heart rate–related features may also reflect broader contextual factors such as habitual physical activity, sleep debt, illness, academic or work schedules, weekend social activity, shift work, or device wear time. Future studies incorporating more granular contextual information and person-centered deviations from individual baselines will be needed to clarify which wearable-derived changes are most specific to alcohol-related risk.

These findings align with prior work using mobile sensor data, which demonstrated that increased physical movement—such as greater acceleration and travel distance—preceded binge-drinking episodes by 1-6 hours [11]. Other studies have similarly reported that fluctuations in activity levels serve as meaningful predictors of drinking behavior [10,12], supporting the relevance of activity-related digital signals as predictive features for drinking-related outcomes. Although this study focused on weekly episode prediction rather than immediate predrinking states, the finding that activity- and calorie-related wearable summaries contributed to model prediction is directionally consistent with previous work on sensor-based alcohol-risk prediction. However, these similarities should be interpreted cautiously because this study did not directly measure immediate predrinking states and cannot determine whether the observed wearable patterns reflected drinking-related processes or other contextual factors. Prior research has also shown that individuals who drink tend to be more physically active than nondrinkers [39], and population-based health surveillance data in Korea likewise indicate that among adults aged 20-39 years, higher levels of alcohol consumption are associated with greater physical activity [40].

A notable finding was that calorie-related variables contributed strongly to model predictions in both integrated models and differed between the high-risk episode and nonepisode groups. Unlike heart rate or step count, both of which largely reflect gross physical movement, calorie expenditure is a composite wearable-derived metric that may be influenced by sleep-wake rhythm, arousal level, metabolic factors, device estimation algorithms, and the intensity and quality of physical activity. However, this pattern should not be interpreted as evidence of altered metabolic efficiency or disrupted biological rhythms. Rather, it indicates that calorie-related wearable summaries were useful predictive features in this model. Although prior research has suggested that alcohol use may be associated with disruptions in biological rhythms [41], this observational sensing data cannot determine whether the observed calorie-related patterns reflected alcohol-related physiological processes or other contextual factors. Because calorie estimates from consumer wearables can be affected by activity type, device wear time, individual physiology, and algorithmic estimation error, this finding should be interpreted cautiously and validated in future studies using more granular contextual and physiological data.

Taken together, the findings suggest that integrating wearable-derived physiological signals with psychological and personality-based vulnerability factors may provide useful information for week-ahead prediction of high-risk drinking episodes. In particular, personality vulnerabilities such as impulsivity and emotional instability, together with activity-, calorie-, and heart rate–related wearable summaries, emerged as predictive correlates of subsequent high-risk drinking labels. These results provide preliminary support for risk-stratified digital monitoring approaches, while also indicating that this model is not yet sufficient as a stand-alone early-warning or intervention-triggering system. Future JITAI-oriented models may require more proximal within-person signals, such as ecological momentary assessment (EMA)–based affective states, individualized deviations from wearable baselines, and contextual data that can distinguish alcohol-related risk signals from broader daily-life variability.

From a translational perspective, these findings are best interpreted as supporting a preliminary risk-stratification approach rather than immediate deployment as a stand-alone early-warning system. A wearable-assisted prediction system of this kind could be most realistically implemented as part of a stepped-care workflow in which model-generated risk signals prompt low-burden self-monitoring feedback, brief motivational messages, or clinician review when elevated risk persists. In primary care, community mental health, university counseling, or addiction service settings, such a system could support clinicians, counselors, or care coordinators in identifying young adults who may benefit from closer monitoring or preventive intervention. This implementation pathway is consistent with evidence suggesting that practitioner-delivered alcohol interventions may have greater short-term effects than digitally delivered interventions alone, while digital interventions may still provide scalable benefits over time and at the population level [42]. Therefore, the model may be most useful when integrated into ongoing professional support or postintervention follow-up, where weekly risk estimates can help guide timely preventive feedback, brief intervention, or referral.

Even when predictive performance remains preliminary, low-burden digital risk feedback may have public health value if it helps individuals recognize periods of elevated vulnerability and prepare preventive coping strategies before high-risk drinking occurs. This aligns with a precautionary public health perspective, in which early, proportionate, and low-risk preventive actions may be justified when potential harms are substantial, and the intervention burden is minimal [43]. However, responsible deployment would require higher and externally validated predictive performance, clear escalation pathways, user consent, transparent alert logic, and safeguards to prevent unnecessary alarm, stigma, or overintervention. In this regard, the model may be better suited to an institution- or service-mediated business to business to consumer–type model than to a stand-alone direct-to-consumer application [44]. In such a model, individuals would use the mobile app and wearable device in daily life, while health care providers, counseling services, public health agencies, university counseling centers, workplace mental health programs, or addiction care coordinators would provide oversight, interpret risk signals, and connect users to appropriate support when needed. In the South Korean context, this approach would also require careful attention to privacy, data minimization, secure storage, governance of sensitive behavioral and health data, regulatory classification, and clear rules regarding who is authorized to access and act on model-generated risk information.

### Limitations

Despite the meaningful findings of this study, several limitations should be acknowledged. First, the sample consisted exclusively of young adults in their 20s, which constrains the generalizability of the results. High-risk drinking patterns are known to vary substantially across age groups, occupational contexts, and sociocultural environments; therefore, the predictive performance demonstrated in this cohort may not translate directly to older adults or clinical populations with more severe alcohol-related problems. Nevertheless, given the rising prevalence of problematic drinking among young adults in South Korea [1,2], early detection and intervention efforts targeting this age group carry important preventive value. Future studies should expand the model to a broader age range to evaluate its robustness across more diverse populations.

Second, this study developed prediction models using relatively short-term longitudinal data from 5 assessment time points over a 4-week follow-up period. As a result, the models were unable to capture long-term fluctuations in drinking behavior, seasonal or contextual influences, and broader temporal dynamics that may affect alcohol use risk. Future work should incorporate longer monitoring periods to more precisely characterize how changes in biosignal patterns interact with shifts in drinking risk over time. Although certain mental health conditions, such as mood or anxiety disorders, often show seasonal or cyclical patterns and therefore require extended longitudinal observation [28,30], such temporal regularity is not a defining feature of addictive behaviors. Indeed, prior studies have reported that using only 1-3 days of past smartphone sensor data was sufficient to enhance model performance for predicting drinking episodes [12]. From an implementation standpoint, accumulating excessively long digital histories may not be advantageous. In real-world mobile apps, storing and processing large volumes of behavioral and physiological data can impose substantial data usage burdens, reduce algorithmic efficiency, and negatively affect user adherence [45]. Thus, while long-term data collection can provide analytic benefits, it may conflict with the practical requirement for lightweight, low-burden, and scalable digital health solutions.

Third, psychological and personality vulnerability variables were measured only at baseline and were therefore modeled as time-invariant predictors. As a result, these variables likely captured between-person differences in underlying vulnerability rather than within-person psychological changes preceding a high-risk drinking episode. Although wearable-derived weekly summaries provided time-varying behavioral and physiological information, this model did not directly estimate individualized deviations from each participant’s own psychological baseline. This distinction is important for interpreting the model as a week-ahead risk prediction tool. Emotional states and stress levels can fluctuate considerably on a week-to-week basis, and treating these variables as static may have influenced both predictive performance and interpretability. The findings suggest that stable vulnerability profiles can improve risk stratification when combined with weekly wearable signals, but they should not be interpreted as demonstrating that baseline psychological variables function as proximal early-warning signals.

However, the psychological measures used in this study, including depression (PHQ-9), anxiety (GAD-7), perceived stress (PSS-10), and personality vulnerability indicators, were originally developed to capture relatively stable symptom severity or vulnerability profiles rather than rapid state-level changes [32,34,46]. Consequently, although repeated weekly self-report symptom scores may provide additional predictive information, they differ conceptually from the baseline vulnerability indicators used in the primary models. The primary modeling framework, therefore, treated psychological and personality measures as baseline vulnerability indicators and focused on whether week-varying wearable-derived summaries could contribute to the prediction of the following week’s high-risk drinking label.

Nevertheless, research using EMA for affective disorders has demonstrated that negative affect often shows substantial moment-to-moment fluctuation, whereas positive affect tends to exhibit dampened diurnal variation [47,48]. Emotional states also react strongly to daily stressors [49]. Therefore, future models should incorporate EMA-based affective states, repeated brief affect measures, or person-centered deviation features to better capture within-person changes that may be more actionable for JITAI delivery.

Fourth, because this was an observational sensing study, wearable-derived predictors may have been influenced by unmeasured contextual and behavioral factors. For example, physical activity routines, sleep debt, acute illness, academic or work schedules, weekend social activity, shift work, device wear time, and other daily-life contexts may affect heart rate, sleep, step count, and calorie estimates independently of alcohol-risk processes. Therefore, SHAP-identified features should be interpreted as predictive correlates rather than causal mechanisms or physiological precursors. Future studies should incorporate contextual measures, EMA-based state assessments, and person-centered deviation features to better distinguish alcohol-related risk signals from broader daily-life variability.

Fifth, the prediction model in this study defined weekly high-risk drinking episodes using a binary threshold based on the AUDIT-K. Because AUDIT-K scores rely on self-reported information, they are subject to recall bias and social desirability bias, which may limit the precision of the outcome labels. It is possible that the model’s predictive performance would differ if more granular event-level drinking assessments or biosensing-based alcohol detection technologies were used.

At the same time, many machine learning and AI studies aiming to predict substance use or alcohol-related risk rely on self-reported labels for the outcome variable [10-12,28,30,50]. Although the reliability of such outcome labels may be debated, they remain the most practical and widely adopted approach in real-world digital phenotyping research. Nonetheless, future studies will benefit from incorporating passive and low-burden outcome measurement tools—such as continuous biosensing devices or ecological momentary detection of drinking events—which may enhance labeling accuracy and further improve predictive performance.

Sixth, although participant-level grouped cross-validation was used to reduce subject-level leakage, the number of positive high-risk drinking episodes within each held-out fold was relatively small. This may have contributed to variability in sensitivity and precision estimates across folds. In addition, the sensitivity-oriented operating threshold improved detection of positive episodes but resulted in modest precision/PPV, indicating that the model may generate false-positive alerts if applied as a stand-alone early-warning tool. Therefore, this model should be interpreted as a preliminary risk-stratification approach rather than a clinically deployable decision system.

Finally, the predictive modeling in this study was limited to 2 tree-based algorithms, XGBoost and random forest. These models were chosen to balance interpretability and predictive performance, particularly given our focus on identifying the likelihood of entering the high-risk drinking category in the following week. However, tree-based models have only limited capacity to capture temporal dependencies or long-range sequential patterns inherent in week-to-week behavioral fluctuations. Future research should incorporate more advanced time-series deep learning approaches, such as long short-term memory networks, temporal convolutional networks, or transformer-based architectures, which may better model temporal dynamics and improve prediction accuracy as longer-term longitudinal data become available.

### Conclusions

This study developed machine learning models integrating emotional and personality vulnerability factors with wearable-derived physiological and behavioral signals to predict weekly high-risk drinking episodes among young adults. The findings suggest that combining stable baseline vulnerability indicators with week-level wearable summaries may provide useful information for week-ahead risk estimation. Activity-, sleep-, heart rate-, and vulnerability-related features contributed to model prediction, although these features should be interpreted as predictive correlates rather than causal mechanisms or proximal physiological precursors.

Importantly, this work provides preliminary evidence for the feasibility of wearable-assisted risk stratification for alcohol-related risk in South Korea. Rather than establishing a fully deployable early-warning system, these findings offer an initial basis for future digital monitoring approaches. Future studies involving broader age groups, longer-term longitudinal data, more proximal within-person measures, and external validation will be essential to enhance the generalizability, clinical use, and JITAI relevance of prediction models for high-risk drinking.

## Data Availability

The datasets generated and analyzed during this study contain sensitive personal health and behavioral information and are therefore not publicly available. Deidentified data may be made available from the corresponding author upon reasonable request and with appropriate institutional approval.

## Appendix

Multimedia Appendix 1 Exploratory model performance when weekly self-report scores were included.

Multimedia Appendix 2 Optuna hyperparameter search spaces.

## Abbreviations

AI: artificial intelligence
AUDIT-K: Alcohol Use Disorders Identification Test-Korean version
EMA: ecological momentary assessment
GAD-7: Generalized Anxiety Disorder-7
JITAI: just-in-time adaptive intervention
NPV: negative predictive value
PHQ-9: Patient Health Questionnaire-9
PPV: positive predictive value
PR AUC: area under the precision-recall curve
PSS-10: Perceived Stress Scale-10
ROC AUC: area under the receiver operating characteristic curve
SHAP: Shapley additive explanations
TRIPOD+AI: Transparent Reporting of a Multivariable Prediction Model for Individual Prognosis or Diagnosis Plus Artificial Intelligence
XGBoost: extreme gradient boosting

## References

[1] Rim SJ, Hahm BJ, Seong SJ, Park JE, Chang SM, Kim BS, An H, Jeon HJ, Hong JP, Park S (2023). Prevalence of mental disorders and associated factors in Korean adults: National Mental Health Survey of Korea 2021. Psychiatry Investig.

[2] Korea Disease Control and Prevention Agency (2023). Community Health Survey Report.

[3] Ministry of Agriculture Food and Rural Affairs (2020). Liquor Industry Information Survey.

[4] Avery AR, Tsang S, Seto EYW, Duncan GE (2020). Stress, anxiety, and change in alcohol use during the COVID-19 pandemic: findings among adult twin pairs. Front Psychiatry.

[5] Giansanti D (2021). The role of the mHealth in the fight against the COVID-19: successes and failures. Healthcare (Basel).

[6] Ryan J, Edney S, Maher C (2019). Anxious or empowered? A cross-sectional study exploring how wearable activity trackers make their owners feel. BMC Psychol.

[7] Cormack F, McCue M, Taptiklis N, Skirrow C, Glazer E, Panagopoulos E, van Schaik TA, Fehnert B, King J, Barnett JH (2019). Wearable technology for high-frequency cognitive and mood assessment in major depressive disorder: longitudinal observational study. JMIR Ment Health.

[8] Nadal C, Earley C, Enrique A, Vigano N, Sas C, Richards D, Doherty G (2021). Integration of a smartwatch within an internet-delivered intervention for depression: protocol for a feasibility randomized controlled trial on acceptance. Contemp Clin Trials.

[9] Gomes N, Pato M, Lourenço AR, Datia N (2023). A survey on wearable sensors for mental health monitoring. Sensors (Basel).

[10] Bae S, Chung T, Ferreira D, Dey AK, Suffoletto B (2018). Mobile phone sensors and supervised machine learning to identify alcohol use events in young adults: implications for just-in-time adaptive interventions. Addict Behav.

[11] Bae SW, Suffoletto B, Zhang T, Chung T, Ozolcer M, Islam MR, Dey AK (2023). Leveraging mobile phone sensors, machine learning, and explainable artificial intelligence to predict imminent same-day binge-drinking events to support just-in-time adaptive interventions: algorithm development and validation study. JMIR Form Res.

[12] Bae S, Ferreira D, Suffoletto B, Puyana JC, Kurtz R, Chung T, Dey AK (2017). Detecting drinking episodes in young adults using smartphone-based sensors. Proc ACM Interact Mob Wearable Ubiquitous Technol.

[13] Hassanpour S, Tomita N, DeLise T, Crosier B, Marsch LA (2019). Identifying substance use risk based on deep neural networks and Instagram social media data. Neuropsychopharmacology.

[14] Guggenmos M, Schmack K, Veer IM, Lett T, Sekutowicz M, Sebold M, Garbusow M, Sommer C, Wittchen H, Zimmermann US, Smolka MN, Walter H, Heinz A, Sterzer P (2020). A multimodal neuroimaging classifier for alcohol dependence. Sci Rep.

[15] Kamarajan C, Ardekani BA, Pandey AK, Kinreich S, Pandey G, Chorlian DB, Meyers JL, Zhang J, Bermudez E, Stimus AT, Porjesz B (2020). Random forest classification of alcohol use disorder using fMRI functional connectivity, neuropsychological functioning, and impulsivity measures. Brain Sci.

[16] Menon S, Swathi J, Anit S, Nair A, Sarath S (2019). Driver face recognition and sober drunk classification using thermal images.

[17] Lannoy S, Duka T, Carbia C, Billieux J, Fontesse S, Dormal V, Gierski F, López-Caneda E, Sullivan EV, Maurage P (2021). Emotional processes in binge drinking: a systematic review and perspective. Clin Psychol Rev.

[18] Ning K, Gondek D, Patalay P, Ploubidis GB (2020). The association between early life mental health and alcohol use behaviours in adulthood: a systematic review. PLoS One.

[19] D'Aquino S, Kumar A, Riordan B, Callinan S (2024). Long-term effects of alcohol consumption on anxiety in adults: a systematic review. Addict Behav.

[20] Weckesser LJ, Pilhatsch M, Muehlhan M, Endraß T, Miller R (2025). A systematic review with meta-analysis on the relation between acute stress, alcohol consumption and cortisol levels in individuals with a personal, familial or no alcohol use disorder. Transl Psychiatry.

[21] Pedersen DE (2019). Gender differences in college binge drinking: examining the role of depression and school stress. Soc Sci J.

[22] Adan A, Forero DA, Navarro JF (2017). Personality traits related to binge drinking: a systematic review. Front Psychiatry.

[23] Galen LW, Henderson MJ, Whitman R (1997). The utility of novelty seeking, harm avoidance, and expectancy in the prediction of drinking. Addict Behav.

[24] Koller G, Zill P, Skoruppa T, Bondy B, Preuss U, Soyka M (2008). Low level of harm avoidance is associated with serotonin transporter functional haplotype in alcohol-dependent individuals. Psychiatr Genet.

[25] Johansson A, Hansen S (2002). Novelty seeking and harm avoidance in relation to alcohol drinking in intact rats and following axon-sparing lesions to the amygdala and ventral striatum. Alcohol Alcohol.

[26] McNamara IA, King SE, Corbin WR, Fromme K (2022). A longitudinal examination of relations between competitive athletic participation, drinking norms, impulsivity, and sensation seeking and binge drinking throughout college. Psychol Addict Behav.

[27] Sliedrecht W, Roozen HG, Witkiewitz K, de Waart R, Dom G (2021). The association between impulsivity and relapse in patients with alcohol use disorder: a literature review. Alcohol Alcohol.

[28] Lee HJ, Cho CH, Lee T, Jeong J, Yeom JW, Kim S, Jeon S, Seo JY, Moon E, Baek JH, Park DY, Kim SJ, Ha TH, Cha B, Kang H, Ahn Y, Lee Y, Lee J, Kim L (2022). Prediction of impending mood episode recurrence using real-time digital phenotypes in major depression and bipolar disorders in South Korea: a prospective nationwide cohort study. Psychol Med.

[29] Aqajari SAH, Labbaf S, Tran PH, Nguyen B, Mehrabadi MA, Levorato M (2023). Context-aware stress monitoring using wearable and mobile technologies in everyday settings. arXiv. Preprint posted online on December 14, 2023.

[30] Cho CH, Lee T, Kim MG, In HP, Kim L, Lee HJ (2019). Mood prediction of patients with mood disorders by machine learning using passive digital phenotypes based on the circadian rhythm: prospective observational cohort study. J Med Internet Res.

[31] Joe KH, Chai SH, Park AR, Lee HK, Shin IH, Min SH (2009). Optimum cut-off score for screening of hazardous drinking using the Korean version of Alcohol Use Disorder Identification Test (AUDIT-K). J Korean Acad Addict Psychiatry.

[32] Spitzer RL, Kroenke K, Williams JB (1999). Validation and utility of a self-report version of PRIME-MD: the PHQ primary care study. Primary care evaluation of mental disorders. Patient Health Questionnaire. JAMA.

[33] An JY, Seo ER, Lim KH, Shin JH, Kim JB (2013). Standardization of the Korean version of screening tool for depression (Patient Health Questionnaire-9, PHQ-9). J Korean Soc Biol Ther Psychiatry.

[34] Spitzer RL, Kroenke K, Williams JBW, Lowe B (2006). A brief measure for assessing Generalized Anxiety Disorder: the GAD-7. Arch Intern Med.

[35] Seo J, Park S (2015). Validation of the Generalized Anxiety Disorder-7 (GAD-7) and GAD-2 in patients with migraine. J Headache Pain.

[36] Park JH, Seo YS (2010). Validation of the perceived stress scale (PSS) on samples of korean university students. Korean J Psychol Gen.

[37] Korean Psychological Association (2021). Enhancing the precision psychological testing system and improving the overall psychological testing regime [Report in Korean]. Military Manpower Administration.

[38] Marcon G, de Ávila Pereira F, Zimerman A, da Silva BC, von Diemen L, Passos IC, Recamonde-Mendoza M (2021). Patterns of high-risk drinking among medical students: a web-based survey with machine learning. Comput Biol Med.

[39] Piazza-Gardner AK, Barry AE (2012). Examining physical activity levels and alcohol consumption: are people who drink more active?. Am J Health Promot.

[40] Oh S (2023). Association between alcohol drinking status and physical activity in Korean adults using the Community Health Survey. J Korea Contents Assoc.

[41] el-Guebaly N (1987). Alcohol, alcoholism, and biological rhythms. Alcohol Clin Exp Res.

[42] Beyer FR, Kenny RPW, Johnson E, Caldwell DM, Garnett C, Rice S, Simpson J, Angus C, Craig D, Hickman M, Michie S, Kaner EFS (2023). Practitioner and digitally delivered interventions for reducing hazardous and harmful alcohol consumption in people not seeking alcohol treatment: a systematic review and network meta-analysis. Addiction.

[43] Goldstein BD (2001). The precautionary principle also applies to public health actions. Am J Public Health.

[44] Grustam AS, Vrijhoef HJM, Cordella A, Koymans R, Severens JL (2017). Care coordination in a business-to-business and a business-to-consumer model for telemonitoring patients with chronic diseases. Int J Care Coord.

[45] Onnela J (2021). Opportunities and challenges in the collection and analysis of digital phenotyping data. Neuropsychopharmacology.

[46] Cohen S, Spacapan S, Oskamp S (1988). Perceived stress in a probability sample of the United States. The Social Psychology of Health.

[47] Peeters F, Berkhof J, Delespaul P, Rottenberg J, Nicolson NA (2006). Diurnal mood variation in major depressive disorder. Emotion.

[48] Murray G (2007). Diurnal mood variation in depression: a signal of disturbed circadian function?. J Affect Disord.

[49] Suls J, Green P, Hillis S (1998). Emotional reactivity to everyday problems, affective inertia, and neuroticism. Pers Soc Psychol Bull.

[50] Epstein DH, Tyburski M, Kowalczyk WJ, Burgess-Hull AJ, Phillips KA, Curtis BL, Preston KL (2020). Prediction of stress and drug craving ninety minutes in the future with passively collected GPS data. NPJ Digit Med.

