# Supplementary material for: Week-Ahead Prediction of High-Risk Drinking Episodes Among Young Adults Using Wearable Biosignals and Psychological Vulnerabilities: Prospective Observational Machine Learning Study
Source: JMIR Mhealth Uhealth. 2026 Jul 10;14:e88223. doi: 10.2196/88223 (PMC13401073; doi:10.2196/88223)
Supplement: Multimedia Appendix 1 [file mhealth_v14i1e88223_app1.docx]

# Multimedia Appendix 1. Exploratory model performance when weekly self-report scores were included

## Supplementary Table

**Supplementary Table 1. Exploratory model performance when repeated weekly self-report symptom scores were included**

| Feature set | Model | Accuracy | Precision/PPV | Recall/Sensitivity | NPV | F1 score | ROC AUC | PR AUC |
| --- | --- | --- | --- | --- | --- | --- | --- | --- |
| Self-report only | XGBoost | 0.285 (0.217) | 0.113 (0.049) | 0.623 (0.352) | 0.618 (0.346) | 0.176 (0.074) | 0.444 (0.123) | 0.155 (0.078) |
| Self-report only | Random Forest | 0.329 (0.062) | 0.145 (0.058) | 0.785 (0.162) | 0.885 (0.066) | 0.242 (0.085) | 0.524 (0.142) | 0.171 (0.077) |
| Health data only | XGBoost | 0.494 (0.224) | 0.165 (0.054) | 0.639 (0.317) | 0.912 (0.070) | 0.235 (0.079) | 0.655 (0.031) | 0.244 (0.102) |
| Health data only | Random Forest | 0.607 (0.093) | 0.217 (0.095) | 0.657 (0.115) | 0.919 (0.031) | 0.314 (0.104) | 0.663 (0.058) | 0.280 (0.082) |
| Integrated | XGBoost | 0.528 (0.215) | 0.167 (0.079) | 0.585 (0.363) | 0.904 (0.055) | 0.232 (0.103) | 0.668 (0.073) | 0.256 (0.132) |
| Integrated | Random Forest | 0.548 (0.040) | 0.204 (0.073) | 0.799 (0.142) | 0.945 (0.037) | 0.320 (0.096) | 0.716 (0.081) | 0.336 (0.121) |

**Note.** This table presents exploratory analyses that included repeated weekly self-report symptom scores. Weekly self-report scores refer to follow-up PHQ-9, GAD-7, and PSS-10 scores collected during the monitoring period and paired with the subsequent week’s high-risk drinking label when available. These analyses were not the primary modeling approach. They differed from the main models reported in Table 3 in feature definition, available feature–label pairs, and missing-data structure; therefore, their performance estimates should not be directly compared with the primary participant-level grouped cross-validation results. Values are presented as mean (SD) across 5 participant-level grouped cross-validation folds. Threshold-dependent metrics were calculated using a decision threshold of 0.20.

## Supplementary Figures

**A. Wearable-derived health features only, Random Forest**


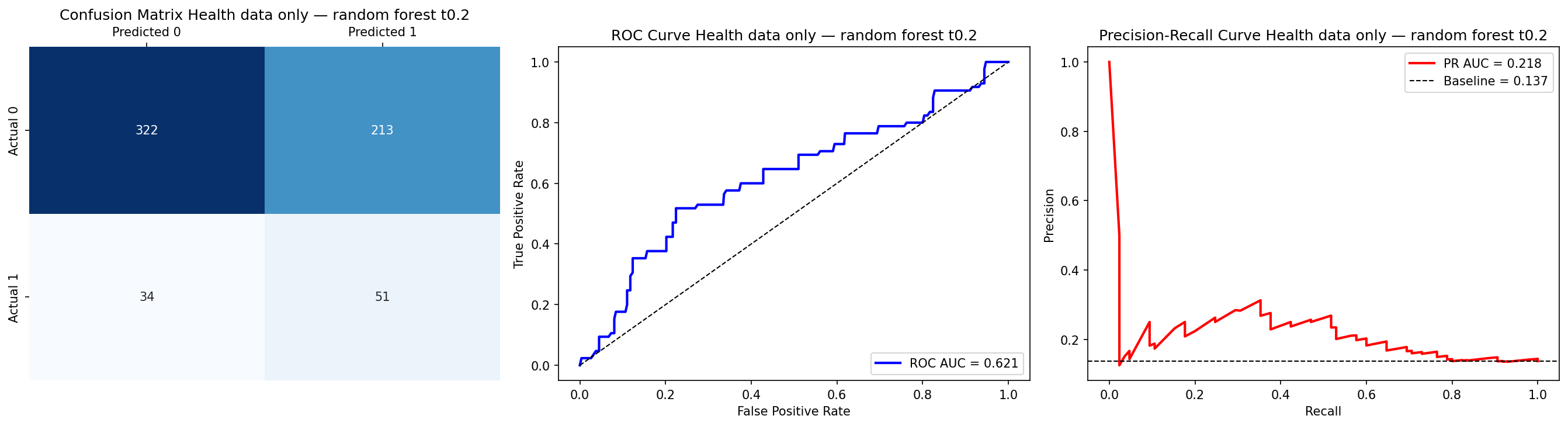


**B. Wearable-derived health features only, XGBoost**


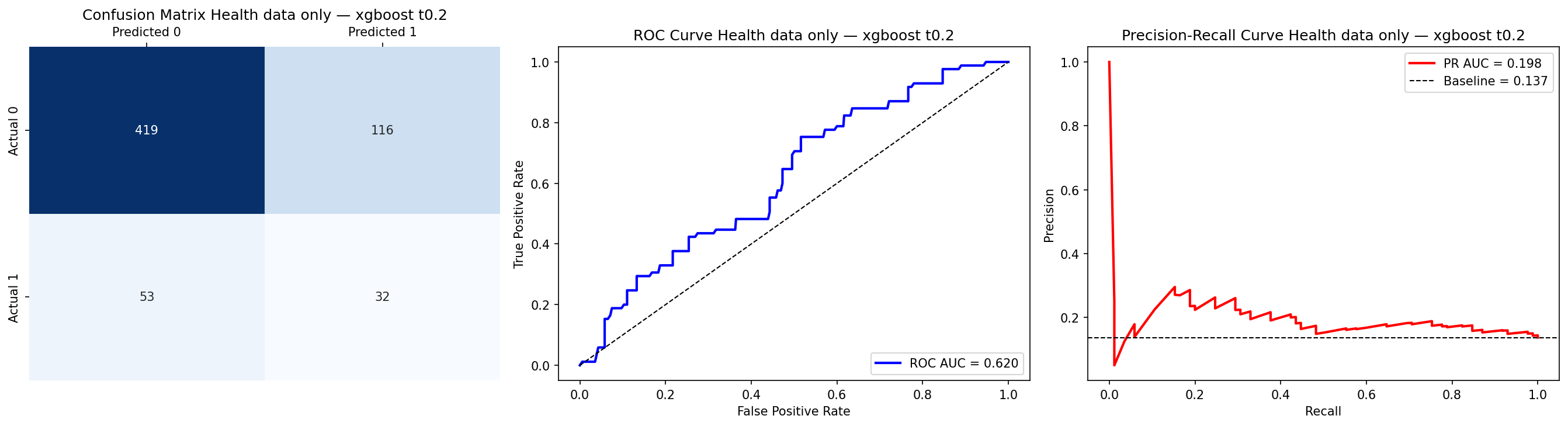


**C. Self-report features only, Random Forest**


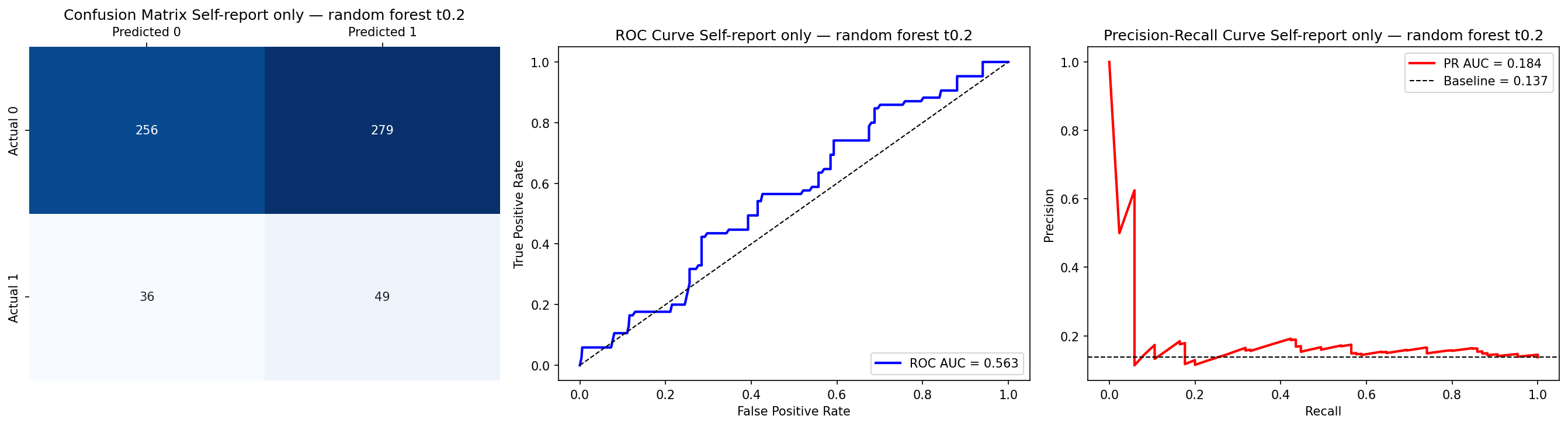


**D. Self-report features only, XGBoost**


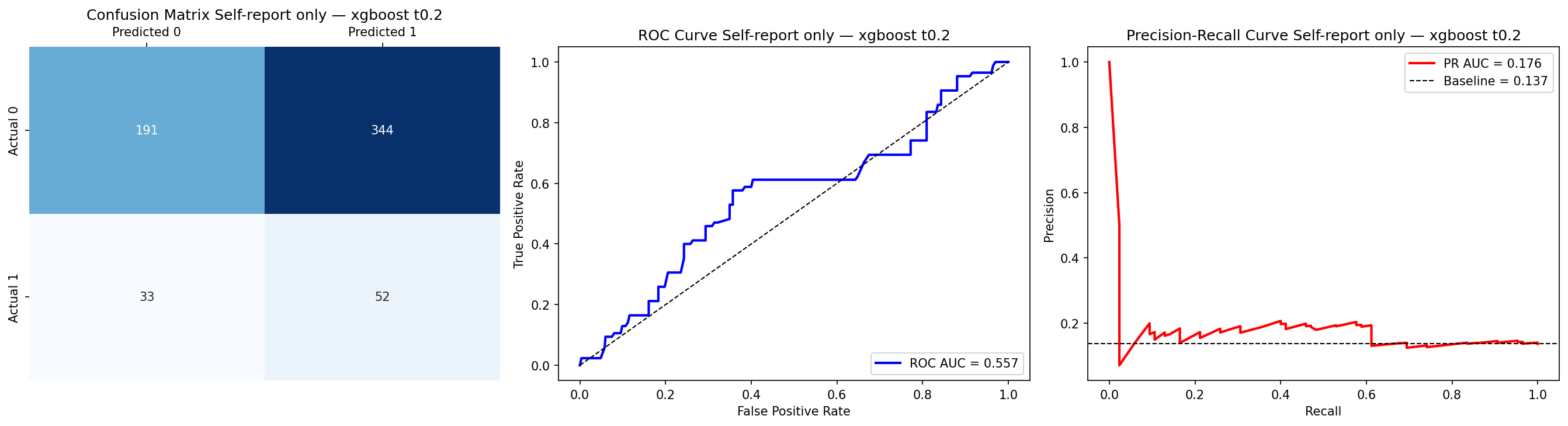


**Supplementary Figure 1. Confusion matrices, ROC curves, and precision-recall curves for single-modality models using pooled out-of-fold predictions at the 0.20 decision threshold**


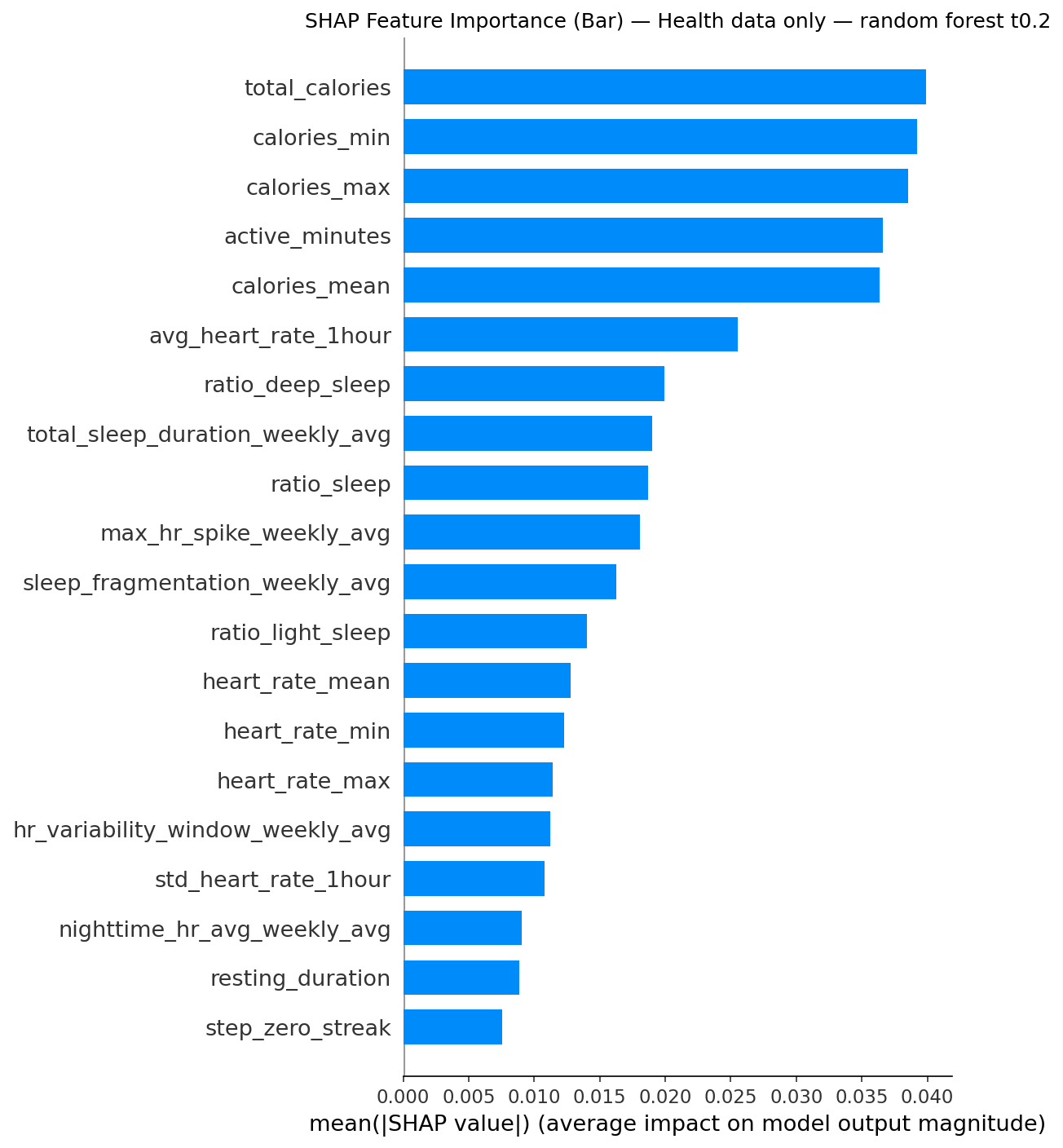

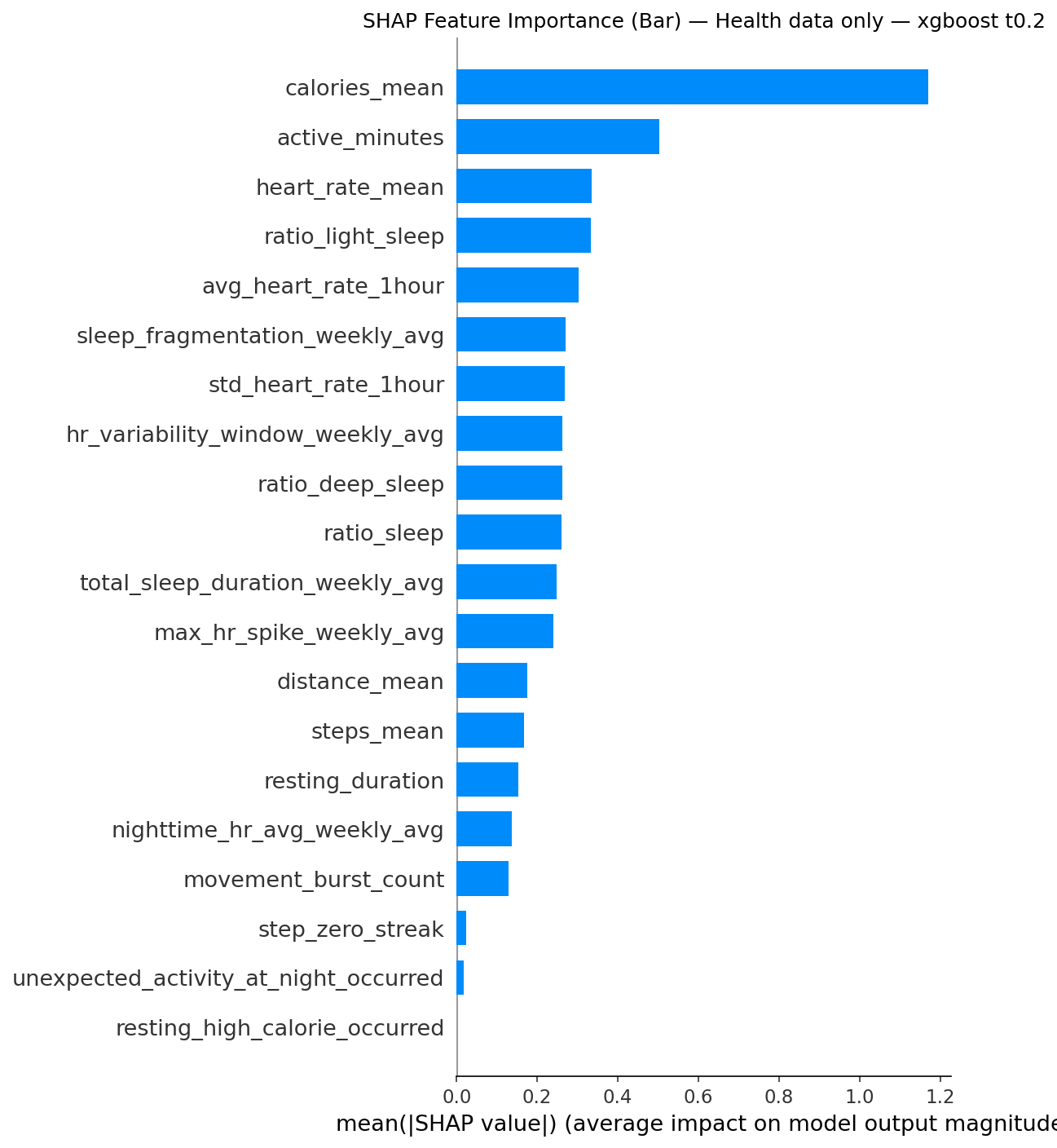


A. Wearable-derived health features only, Random Forest B. Wearable-derived health features only, XGBoost


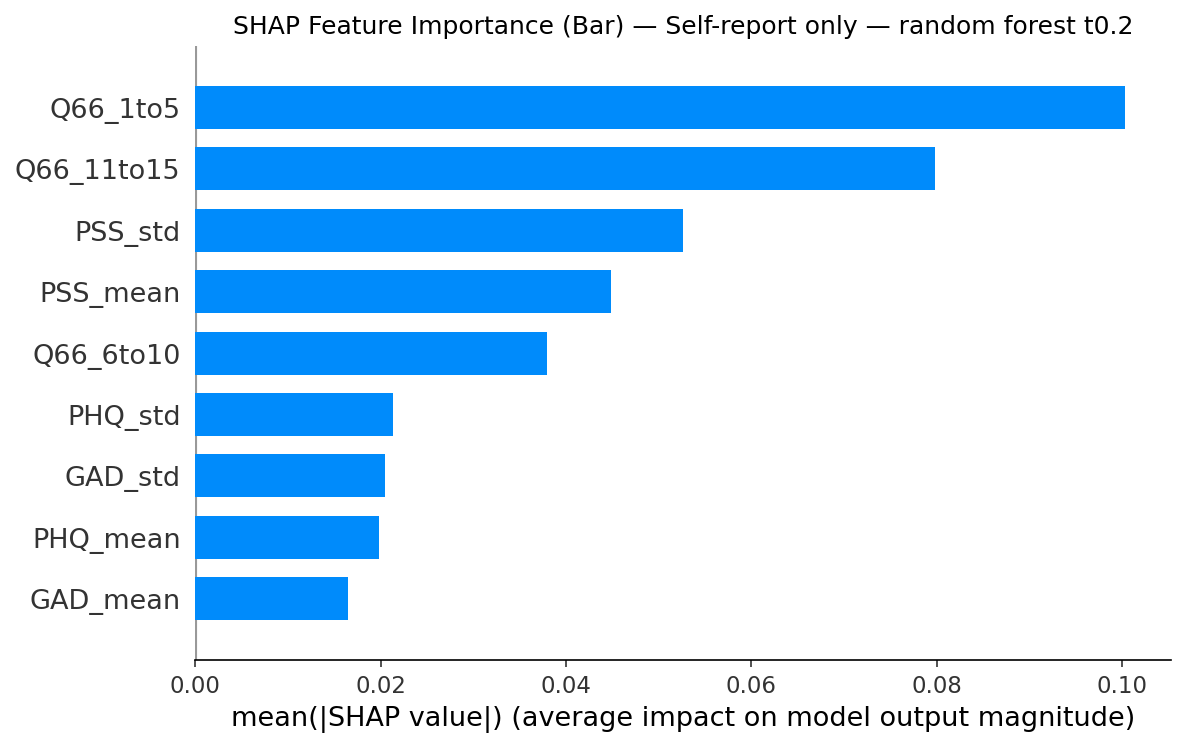

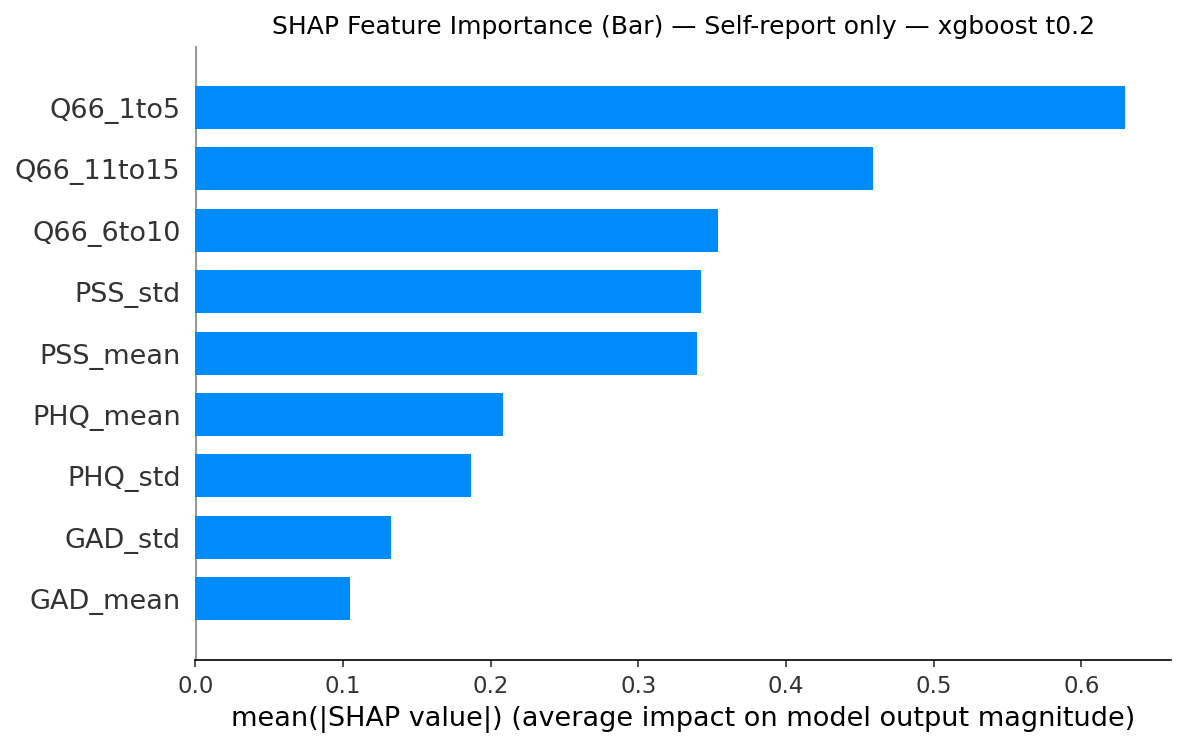


C. Self-report features only, Random Forest D. Self-report features only, XGBoost

Supplementary Figure 2. SHAP feature importance plots for self-report-only and wearable-derived health feature–only models


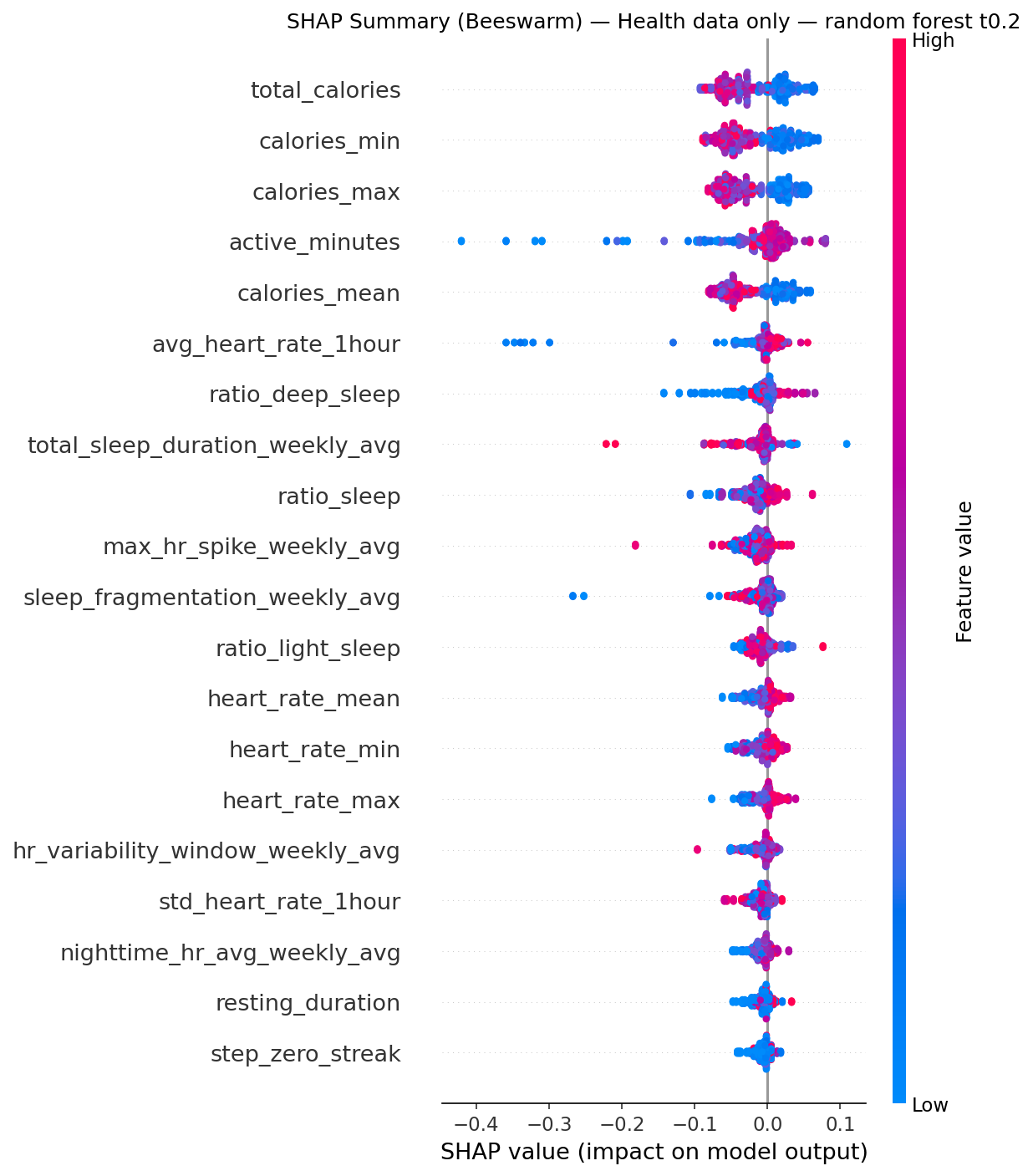

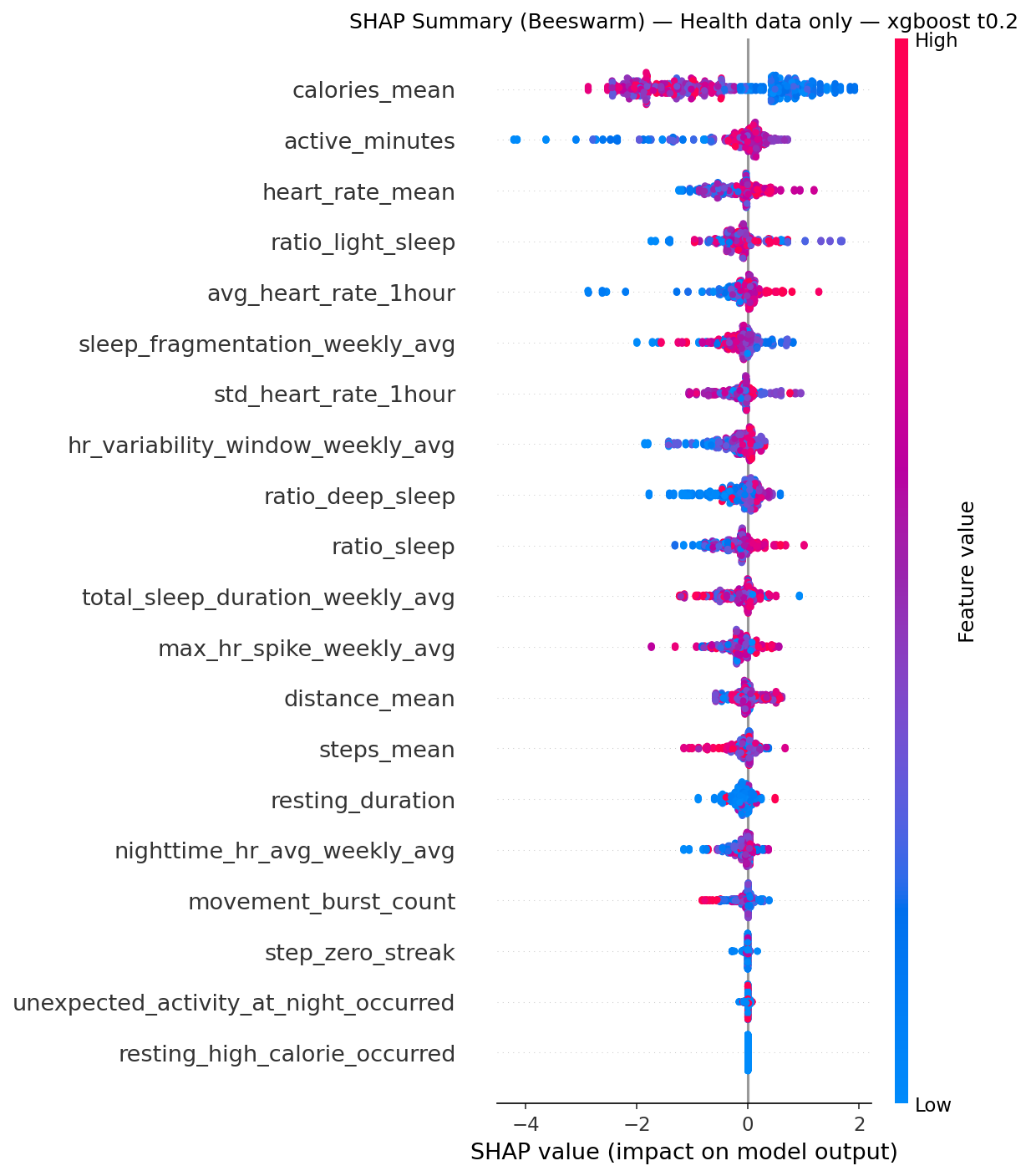


A. Wearable-derived health features only, Random Forest B. Wearable-derived health features only, XGBoost


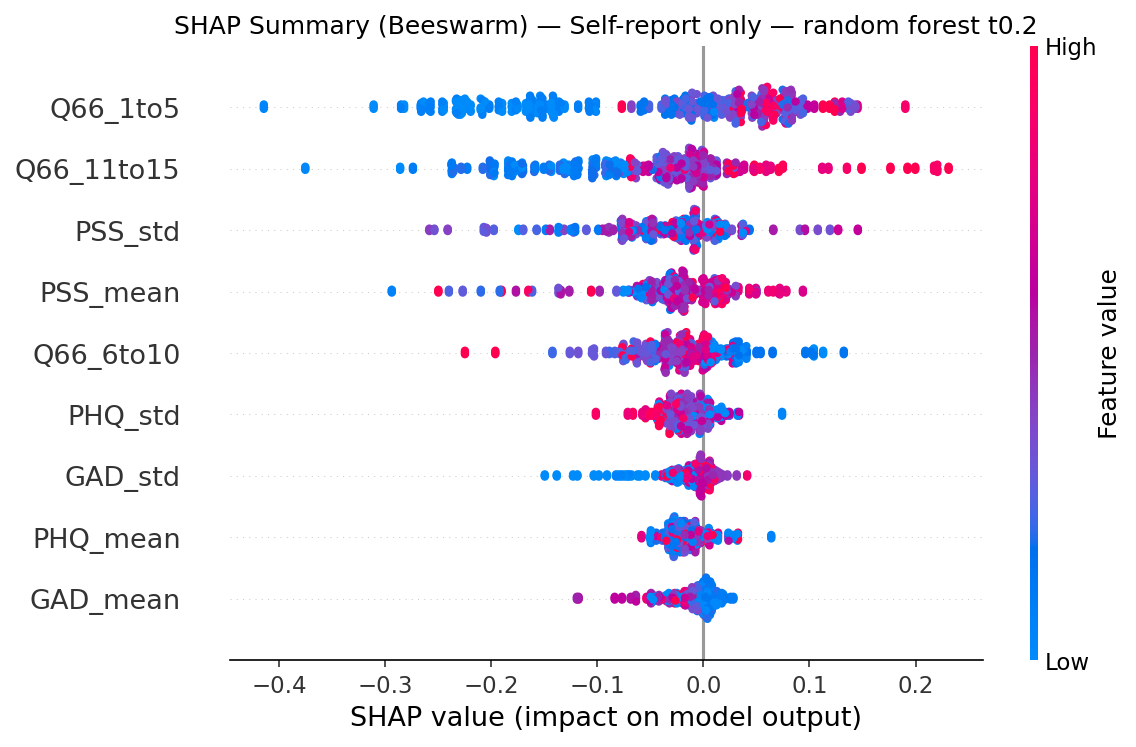

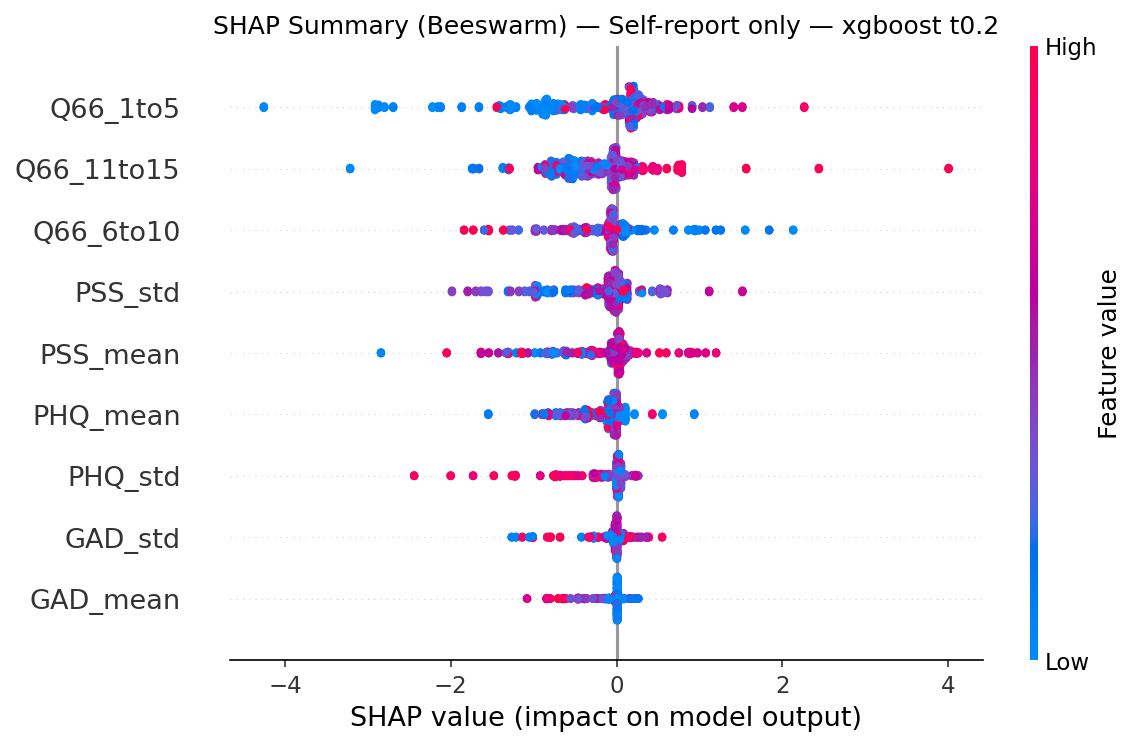


**C. Self-report features only, Random Forest D. Self-report features only, XGBoost**

**Supplementary Figure 3. SHAP summary plots for self-report-only and wearable-derived health feature–only models**
