# Supplementary material for: Week-Ahead Prediction of High-Risk Drinking Episodes Among Young Adults Using Wearable Biosignals and Psychological Vulnerabilities: Prospective Observational Machine Learning Study
Source: JMIR Mhealth Uhealth. 2026 Jul 10;14:e88223. doi: 10.2196/88223 (PMC13401073; doi:10.2196/88223)
Supplement: Multimedia Appendix 2 [file mhealth_v14i1e88223_app2.docx]

# Multimedia Appendix 2. Optuna hyperparameter search spaces

**Supplementary Table 2. Optuna hyperparameter search spaces**

| Model | Hyperparameter | Search space |
| --- | --- | --- |
| XGBoost | n_estimators | Integer, 100-400 |
| XGBoost | max_depth | Integer, 2-6 |
| XGBoost | learning_rate | Continuous, 0.01-0.10 |
| Random Forest | n_estimators | Integer, 100-400 |
| Random Forest | criterion | Categorical: gini, entropy |
| Random Forest | max_depth | Integer, 3-12 |
| Random Forest | min_samples_split | Integer, 2-20 |
| Random Forest | min_samples_leaf | Integer, 1-10 |
| Random Forest | max_features | Categorical: sqrt, log2, None |

**Note**. Optuna tuning was conducted separately within the training portion of each cross-validation fold for each model and feature set. Each tuning run used 100 trials. For XGBoost, scale_pos_weight was calculated from the distribution of the training data within each fold. For Random Forest, class_weight was set to balanced. The held-out fold was not used for hyperparameter selection, model selection, or threshold determination. Model-level random states were fixed at 42.
